# Associations between dietary fatty acid patterns and non-alcoholic fatty liver disease in typical dietary population: A UK biobank study

**DOI:** 10.3389/fnut.2023.1117626

**Published:** 2023-02-07

**Authors:** Aowen Tian, Zewen Sun, Miaoran Zhang, Jiuling Li, Xingchen Pan, Peng Chen

**Affiliations:** ^1^Key Laboratory of Pathobiology, Ministry of Education, Jilin University, Changchun, Jilin, China; ^2^Department of Pathology, College of Basic Medical Sciences, Jilin University, Changchun, Jilin, China; ^3^Department of Genetics, College of Basic Medical Sciences, Jilin University, Changchun, Jilin, China; ^4^Department of Molecular Biology, College of Basic Medical Sciences, Jilin University, Changchun, Jilin, China

**Keywords:** NAFLD, dietary pattern, dietary fatty acids, polyunsaturated fatty acids, liver fat content

## Abstract

**Background and Aims:**

Dietary fatty acid composition is associated with non-alcoholic fatty liver disease (NAFLD). Few evidence had identified a clear role of dietary fatty acid composition of typical diet in NAFLD. We aimed to investigate the relationship between dietary patterns and NAFLD in populations with typical diets and to explore the effect of fatty acid composition in dietary patterns on NAFLD.

**Methods:**

Principal component analysis was used to identify 4 dietary patterns in UK Biobank participants. Logistic regression was used to estimate the association between dietary patterns and NAFLD. Mediation analysis was performed to evaluate the extent to which the relationship between dietary patterns and NAFLD was explained by dietary fatty acid combinations, as surrogated by serum fatty acids measured by nuclear magnetic resonance.

**Results:**

A dietary fatty acid pattern (DFP1) characterized by “PUFA enriched vegetarian” was negatively associated with NAFLD risk. Serum fatty acids were significantly associated with DFP1 and NAFLD. Mediation analysis showed SFA (27.8%, *p* < 0.001), PUFA (25.1%, *p* < 0.001), ω-6 PUFA (14.3%, *p* < 0.001), LA (15.6%, *p* < 0.001) and DHA (10%, *p* < 0.001) had a significant indirect effect on the association between DFP1 and NAFLD. A dietary pattern characterized by “PUFA enriched carnivore” (DFP2) was not associated with NAFLD risk.

**Conclusion:**

A “PUFA enriched vegetarian” dietary pattern with increased LA and DHA, may be beneficial for the treatment or prevention of NAFLD, while a “PUFA enriched carnivore” dietary pattern may not be harmful to NAFLD.

## 1. Introduction

The metabolic disease nonalcoholic fatty liver disease (NAFLD) is becoming the most common chronic liver disease worldwide (1). Globally, about 25–30% of adults and about 15% of children develop NAFLD (2). NAFLD increases the risk of further developing liver cirrhosis or hepatocellular carcinoma (HCC) (3, 4). Unhealthy dietary patterns (such as increased caloric intake, especially glucose, fructose, and saturated fat) and sedentary behavior have been shown to increase liver fat content, which has contributed to the development of NAFLD (5).

Dietary intake is considered to be a modifiable risk factor for NAFLD (6). Previous studies have identified that isoenergetic diets with different fatty acid compositions affect the accumulation of hepatic fat differentially (7, 8). The previous results identified the beneficial effects of PUFA and the harmful effects of SFA on NAFLD/hepatic fat (9–11). However, the designed fatty acid intake in most experiments far exceeded typical diets in the real world, resulting in poor application to normal healthy populations (12).

Studies on dietary patterns established according to typical dietary populations may be more beneficial to provide practicable clinical strategies on diet (13). The vast majority of current research studying the role of dietary patterns on NAFLD has been designed using food groups, while only a small number of studies were performed using nutrient intakes (14–16). Using nutrient intakes to identify dietary patterns enables the understanding of key biological processes and makes it easier to compare results between different populations (17). Dietary fat composition is an important manifestation of nutrient-based dietary patterns, but there has been few study revealed the effects of the dietary fat composition of nutrient-based dietary patterns on NAFLD/hepatic fat content. Addressing the effects of fatty acid composition in different dietary patterns on NAFLD will promote the development of dietary treatment strategies for NAFLD. A previous study has revealed the fatty acid composition in serum as a useful marker for dietary fatty acid intake (18), making it feasible to measure the effect of dietary fatty acid composition on NAFLD in large cohorts.

Here, we performed nutrient-based dietary pattern analysis using the UK Biobank cohort to investigate the association between dietary fatty acid patterns and NAFLD in a typical dietary population. We also test whether serum fatty acids mediate the relationship between dietary fatty acid patterns and NAFLD.

## 2. Methods

### 2.1. Participants and sample exclusion

The UK Biobank (UKB) cohort comprised more than 500,000 participants aged 40–69 from the UK population during 2006–2010 (19). These participants provided extensive genetic and phenotypic data. The UKB collected dietary information in about 210,000 participants through a web-based 24-h recall questionnaire, Oxford WebQ^1^ (20).Participants with atypical diet, unreliable energy intake (<500 or > 3,500 kcal/day for women and < 800 or > 4,000 kcal/day for men), incomplete phenotype (nutrients, BMI, income, and education), HBV/HCV infection, liver-related disease (hemochromatosis, viral hepatitis, Wernicke’s disease, and Wilson disease), liver damage drugs use, alcohol use and alcoholic diseases were excluded (Supplementary Table S1). Ultimately 93,399 unrelated individuals of European ancestry were used for further analysis.

### 2.2. Dietary fat intake patterns

The total quantity consumed of each food or beverage for each participant was calculated by multiplying the portion size by the amount consumed per portion. The nutrient intakes were calculated by multiplying the total quantity consumed by the composition of nutrients in the food or beverage. The majority of portion sizes was defined from *Food portion sizes* (21), and nutrient composition in food or beverages was defined from *McCance and Widdowson’s Composition of Foods* (22). Fiber intake was estimated using the Englyst method (23). We calculated MUFA intake by subtracting SFA and PUFA from total fat intake (24).

These participants were invited to complete the Oxford WebQ 5 times between 2009 and 2012, and the average nutrient intake was calculated in participants who completed two or more 24-h dietary assessments. Daily nutrients intake was firstly measured as the average intake per 1,000 kcal of total energy intake. Dietary fat intake depends on other nutrients intake, thus we also included other nutrients in subsequent analysis. Given the high correlation between different nutrient intake phenotypes, we performed a principal component analysis (PCA) for nutrient intake data in the UKB cohort. Nutrients intake phenotypes were grouped into a smaller number of uncorrelated underlying factors, also known as dietary patterns. The number of factors (dietary patterns) was retained based on the following criteria: eigenvalues >1, Cattell scree test, and interpretability of the factors. Varimax rotation was performed to generate uncorrelated and interpretable dietary patterns, which were named according to dietary fat intake characteristics. Factor scores were calculated for each participant, with higher scores closer to the corresponding dietary pattern. To examine the differences in characteristics according to different dietary fat intake patterns (DFPs) quartiles, analysis of variance (continuous data) and logistic regression analyzes (categorical data) were undertaken.

### 2.3. Non-alcoholic fatty liver disease definitions

The whole liver proton density fat fraction (WL-PDFF) was got from magnetic resonance images of 42,891 participants in UK Biobank (UK Biobank project 71,668). WL-PDFF had a high correlation with previous PDFF measurement (25). NAFLD was diagnosed by WL-PDFF ≥ 5%, while healthy controls were defined as WL-PDFF < 5%.

### 2.4. Observational study

Multivariate linear regression analysis was used to examine the cross-sectional association between DFPs and NAFLD, 4 hepatic enzymes (GGT, AST, ALT, and ALP), 6 lipid-related biomarkers (APOA, APOB, TC, TG, LDL-C, and HDL-C) or 247 serum metabolic biomarkers (generated by Nightingale Health and provided by the UKB). Two models have been applied in our analysis: the minimum-adjustment model and the fully-adjusted model. In the minimally adjusted model, we adjusted for age, gender, and BMI; in the fully adjusted model, we further adjusted for sedentary time, exercise, income, and education. Details of the covariate coding can be found in the Supplementary text. FDR *p* < 0.05 was used as the significance level in each model.

### 2.5. Mediation analysis

Mediation analysis was performed to assess whether the effect of DFP on NAFLD was mediated by the serum metabolic biomarkers. The mediation analysis aimed to quantify the effect of exposure on the outcome (natural indirect effect, NIE) mediated by mediating variables (exposure → mediating variable → outcome). In our study, the total effect of DFP on NAFLD was decomposed into estimates of the natural direct effect (NDE) of DFP (not mediated by metabolic biomarkers) and the NIE of DFP (mediated by metabolic biomarkers). The proportion of mediation (PM) was calculated using the following formula (26):


$$\mathrm{PM}=\frac{lnOR_{NIE}}{\left(lnOR_{NIE}+lnOR_{NDE}\right)}\times 100\%$$

Mediator models were adjusted for age, gender, and BMI. For all mediated models, confidence intervals of 95% (95% CI) were calculated by non-parametric bootstrap with 1,000 replications.

### 2.6. Statistical analysis

PCA was performed using the “psych” package of R (version 2.1.6, June 18, 2021) (27). Multivariate logistic/linear regression analyzes were performed using R software (version 4.0.2).^2^ Mediation analysis was performed using the “medflex” package of R (version 0.6-7, August 3, 2020) (28).

## 3. Results

### 3.1. Dietary fat intake patterns in a typical dietary population

**Figure 1:**
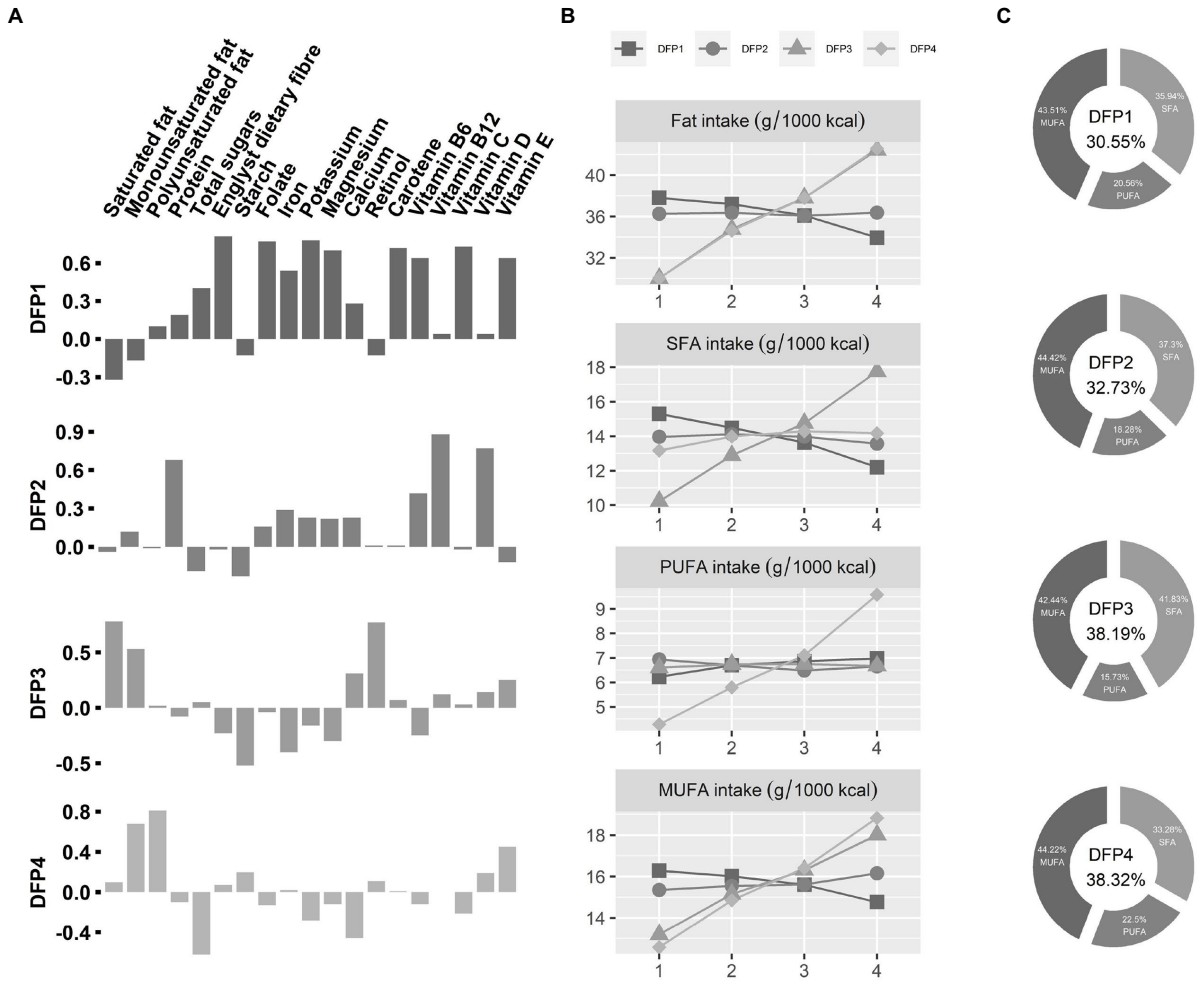
Characteristics of Dietary fat intake patterns (DFPs). **(A)** Loadings of DFPs. The *x*-axis is the various nutrients, and the *y*-axis is the PCA factor loadings of those nutrients. DFPs, Dietary fat intake patterns. **(B)** Dietary fat intake characteristics of DFPs. The *x*-axis is quartiles of DFPs, and the *y*-axis is mean value of dietary fat intake. **(C)** Dietary fatty acid composition for the fourth quartile (highest intake) population. The percentage of total energy from dietary fatty acids was placed in the middle.

Characteristic profiling for the typical dietary participants in UK biobank revealed that the majority of the participants were middle aged (56.8 ± 7.8y), more females than males (52.7% vs. 47.3%), and overweight (average BMI 26.9 ± 4.6 kg/m^2^), with sedentary hours of 3.7 ± 1.9 h per day. Liver fat content was moderate (4.1 ± 3%), with a NAFLD prevalence of 18.3% (Supplementary Table S2). We conducted PCA using a total of 19 nutrients (Supplementary Table S3). Four dietary patterns were identified in typical dietary participants (Supplementary Table S4). These 4 dietary patterns explained 63% of the total variance and were named according to each dietary pattern’s fat intake characteristics (Figure 1). Supplementary Table S5 provides sample characteristics according to quartiles of different DFPs.

DFP1 was defined as “low fat and high PUFA 1,” characterized by low total dietary fat intake (30.55% of total energy) and a high proportion of PUFA (20.56%). The highest intake group (DFP1-Quartile 4) has low SFA and MUFA intake and high PUFA intake compared to the lowest intake group (DFP1-Quartile 1). DFP2 was defined as “low fat and high PUFA 2,” characterized by low total dietary fat intake (32.73% of total energy) and a slightly low proportion of PUFA (18.28%). There was no significant difference in dietary fat between the highest intake group (DFP2-Quartile 4) and the lowest intake group (DFP2-Quartile 1). DFP3 was defined as “high fat and high SFA,” characterized by high total dietary fat intake (38.19% of total energy) and a high proportion of SFA (41.83%). Compared with the lowest intake quartile (DFP3-Quartile 1), the highest intake group (DFP3-Quartile 4) has a high tendency for SFA and MUFA intake, with little change in PUFA intake. DFP4 was defined as “high fat and high PUFA,” characterized by high total dietary fat intake (38.19% of total energy) and a high proportion of PUFA (22.5%). The highest intake group (DFP4-Quartile 4) tended to consume more unsaturated fats than the lowest intake group (DFP4-Quartile1).

### 3.2. Observational association between dietary fat intake patterns and non-alcoholic fatty liver disease

**Figure 2:**
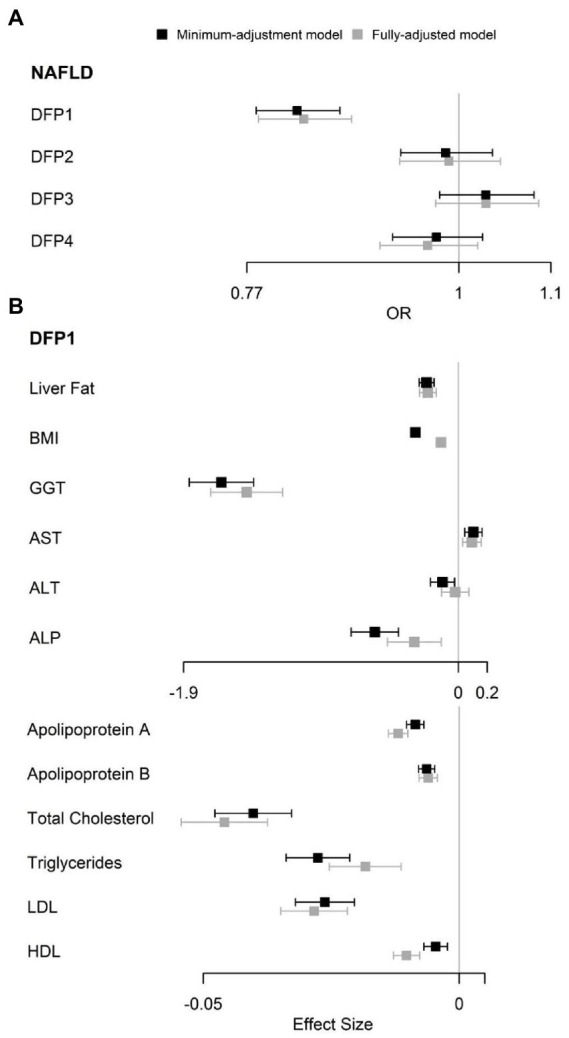
Observational association between DFPs with NAFLD and NAFLD related traits. **(A)** Observational association between DFPs with NAFLD. **(B)** Observational association between DFP1 with NAFLD related traits. OR, odds ratio.

The multivariate logistic regression minimum-adjustment model showed that only DFP1 was significantly associated with NAFLD [OR (95%CI): 0.82 (0.78–0.87), FDR *p* = 2.92 × 10^−11^], and this association remained significant (FDR *p* < 0.05) in the fully-adjusted model (Figure 2A; Supplementary Table S5). Minimum-adjustment multivariate linear models revealed that DFP1 was significantly associated with all 12 NAFLD-related traits included in our study (Supplementary Table S6). DFP1 was positively associated with AST (*β* = 0.1, FDR *p* = 6.93 × 10^−4^), and negatively associated with the rest of NAFLD-related traits (Liver Fat *β* = −0.22; BMI *β* = −0.3; GGT *β* = −1.64; ALT *β* = −0.11; ALP *β* = −0.58; Apolipoprotein A *β* = −0.01; Apolipoprotein B *β* = −0.01; Cholesterol *β* = −0.04; TC *β* = −0.03; LDL-C *β* = −0.03; HDL-C *β* = −0.004). As a sensitivity analysis, we also tested these associations using fully-adjustment multivariate linear models (Supplementary Table S6). All associations remained significant except ALT (*β* = −0.02, FDR *p* = 0.65; Figure 2B).

### 3.3. Observational association between dietary fat intake patterns, non-alcoholic fatty liver disease and serum metabolic biomarkers

**Figure 3:**
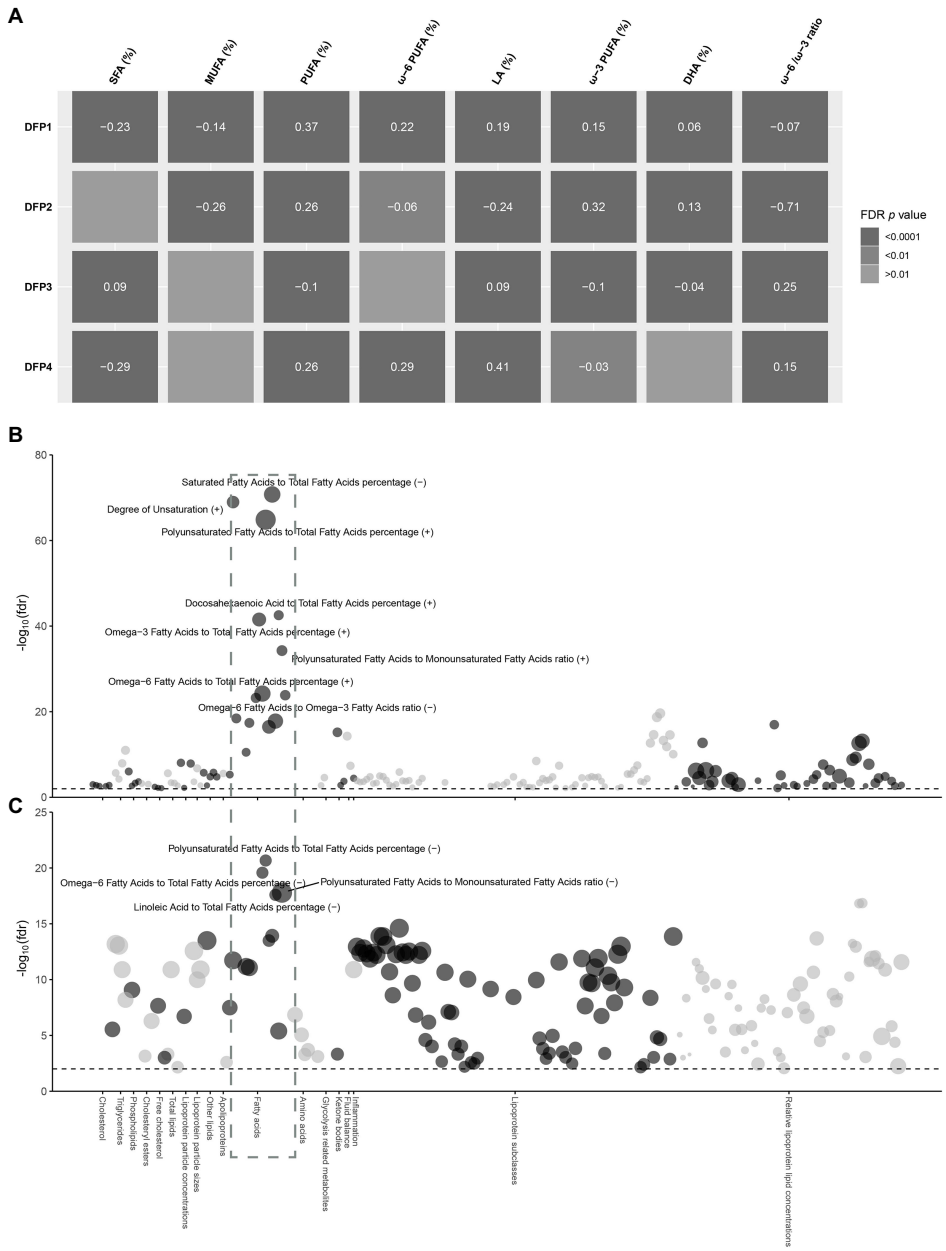
Observational association between DFP1 and NAFLD with serum metabolic biomarkers. **(A)** Heat map of the correlation between DFPs and serum fatty acids. Colors indicate FDR *p* values, the values in the center of the color blocks represent effect sizes, and the positive and negative represent the direction of the effect. **(B)** Observational association between DFP1 with serum metabolic biomarkers. **(C)** Observational association between NAFLD with serum metabolic biomarkers. The radial axis is the significance of association in −log_10_(FDR *p*), and the black dashed line represents the significance level FDR *p* < 0.01. The size of the point represents the size of the effect value, and the positive and negative represent the direction of the effect.

Minimum-adjustment multivariate linear models revealed DFPs were highly correlated with serum fatty acids, consistent with their dietary fatty acid compositions (Figure 3A). DFP1 was negatively correlated with SFA and MUFA, and positively associated with ω-6 PUFA (*β* = 0.22, FDR *p* = 1.98 × 10^−22^) and ω-3 PUFA (*β* = 0.15, FDR *p* = 3.00 × 10^−46^). Interestingly, DFP1 was also negatively correlated with the ω-6 PUFA to ω-3 PUFA ratio. DFP2 was similar to DFP1 in dietary fatty acid composition while different in serum fatty acids association. Although DFP2 was also positively correlated with serum PUFA (*β* = 0.26, FDR *p* = 8.17 × 10^−32^), it was positively correlated with ω-3 PUFA (*β* = 0.32, FDR *p* = 2.11 × 10^−228^) and negatively correlated with ω-6 PUFA (*β* = −0.06, FDR *p* = 8.17 × 10^−3^). DFP3 was positively correlated with serum SFA, which is consistent with the dietary fatty acid composition. The association between DFP4 and serum fatty acids was also consistent with the dietary fatty acid composition, with a negative correlation with serum SFA and a positive association with PUFA.

To further explore the mechanisms in which DFP1 affects NAFLD, we investigated the association between DFP1 and serum metabolic markers (Supplementary Table S7). A total of 188 serum metabolic markers were significantly associated with DFP1, with serum fatty acids being the most significant markers (Figure 3B). We also investigated the association between NAFLD and serum metabolic markers (Supplementary Table S8). A total of 184 serum metabolic markers were significantly associated with NAFLD, with serum fatty acids also being the most significant (Figure 3C). SFA and MUFA were associated with increased risk of NAFLD, while ω-6 PUFA [OR (95%CI): 0.86 (0.84, 0.89), FDR *p* = 2.65 × 10^−20^] and LA [OR (95%CI): 0.86 (0.83, 0.89), FDR *p* = 2.54 × 10^−18^] were correlated with lower risk of NAFLD. No association between ω-3 PUFA and NAFLD was observed, but DHA [OR (95%CI): 0.7 (0.59, 0.82), FDR *p* = 4.04 × 10^−6^] was negatively associated with NAFLD. As a sensitivity analysis, we also analyzed liver fat content rather than NAFLD. Liver fat was significantly positively associated with SFA and MUFA, and negatively associated with ω-6 PUFA, LA, and DHA, which is consistent with the association between NAFLD and serum fatty acids (Supplementary Figure S1). 142 serum metabolic markers including 11 fatty acid markers were significantly associated with both DFP1 and NAFLD (Supplementary Table S9). DFP1-associated serum fatty acids were significantly associated with low liver fat content and NAFLD risk, implicating that DFP1 is likely to protect NAFLD by regulating serum fatty acids.

### 3.4. Mediation analysis of dietary fat intake patterns and non-alcoholic fatty liver disease

**Figure 4:**
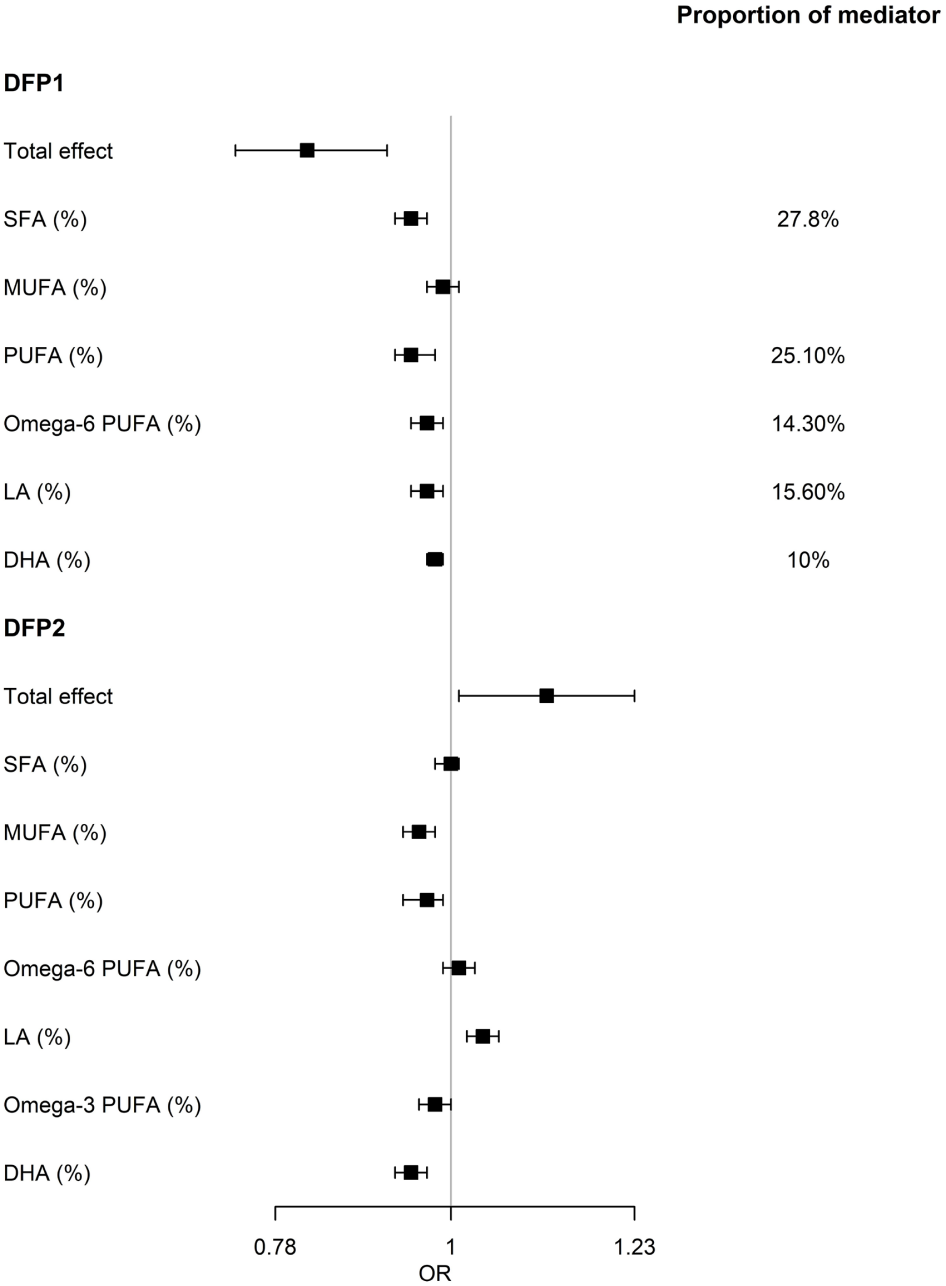
Results of the mediation analysis. Serum fatty acids mediates the relationship between DFPs and NAFLD. OR, odds ratio of mediation effect. Proportion of mediator, proportion of the effect of DFP on NAFLD that goes through fatty acids.

To further explore the causal relationship between DFPs and NAFLD, we used mediation analysis to assess whether the effect of DFP1 on NAFLD was mediated by serum fatty acids. Eleven serum fatty acid markers both significantly associated with DFP1 and NAFLD were included in the mediation analysis. Eight serum fatty acid markers showed an indirect effect (NIE *p* < 0.05, Supplementary Table S10). Among them, SFA, PUFA, ω-6 PUFA, LA, and DHA explained 27.8%, 25.1%, 14.3%, 15.6%, and 10% of the total effect of DFP1 on NAFLD, respectively (Figure 4).

Although the remaining 3 DFPs were not significantly associated with NAFLD, we also used mediation analysis to avoid ignoring the effect of the dietary fat combination on NAFLD due to the masking effect of other nutrients (Supplementary Table S10). In DFP2, only LA showed a marginal significant indirect effect on NAFLD [OR (95%CI): 1.12 (1.01, 1.23), *p* = 0.02], while indirect effects of MUFA, PUFA, ω-3 PUFA, and DHA were not consistent with total effect (Figure 4), implying that the partial effect of DFP2 was masked. No serum fatty acids showed an indirect effect in the association between DFP3/DFP4 and NAFLD.

## 4. Discussion

To our best knowledge, this is the best powered study of the effect of daily dietary patterns on NAFLD, which is diagnosed by precise MRI-PDFF measurements. In this study, typical dietary patterns were inferred from the questionnaire that surveyed in general population, of which the results are better transferable into clinical dietary suggestions. As simple liver steatosis can be reverted by dietary intervention, our result could be informative for future clinical management of NAFLD patients.

Decomposing dietary patterns using principal component analysis has been proven to be successful in revealing the contribution of nutrient components to disease risk (29). In our study, 4 dietary fat patterns were identified, of which 3 patterns (DFP1-3) explained 51% variance of interpersonal food intake and were identical to the patterns identified in a previous study (17).

DFP1 was the only dietary fat pattern associated with a reduced risk of NAFLD. DFP1 is characterized by low total energy intake, low total dietary fat intake and a high proportion of PUFA, consistent with beneficial dietary fatty acid in anti-NAFLD diet (6). DFP1 has shown a good weight loss effect, which is beneficial in reducing inflammation, metabolic disorders, insulin resistance and other adverse effects caused by obesity (30). Furthermore, the significant association of DFP1 with liver enzymes and lipid biomarkers suggests that DFP1 may even be associated with the improvements in liver function. DFP1 can reduce the level of glutathione (GSH) degrading enzyme GGT, which indicates that DFP1 has the potential to increase antioxidant levels through GSH (31). DFP1 was also negatively correlated with the liver injury marker ALT, which also indicated a protective effect on the liver. The blood lipid control ability exhibited by DFP1 may be related to the elevated plasma LDL-C clearance and the improvement of lipid metabolism. Although the specific mechanisms underlying these effects of DFP1 shall be clarified in future studies, the potential mechanisms for anti-NAFLD are discussed in the following.

The source of food may modify the effect of dietary fat on the risk of NAFLD. Vegetarian diet was associated with reduced risk of NAFLD (32–34). In our study, DFP1 and DFP2 were similar in terms of dietary fat. However, DFP1 was associated with reduced NAFLD risk, possibly attributed to the vegetable-origin of the food. In fact, the major nutrients of DFP1, e.g., dietary fiber, potassium, folate, carotene, vitamin C, B6, and E, were mostly enriched in vegetables and fruits (35, 36). The benefit of vegetarian diet is also supported by previous studies from diverse populations (14–16), indicating the generalizability of vegetarian food in the management of NAFLD patients.

Although the benefit of vegetarian diet has been demonstrated, the effects of separate fatty acids remain unclear. Our mediation analysis showed that around one fourth of the beneficial effects of DFP1 could be mediated by PUFA intake, with the assumption that serum PUFA level surrogates dietary PUFA intake (18). PUFA has been shown to reduce liver fat, through the activation of PPARs to promote fatty acid oxidation (37, 38) and anti-inflammatory effects (39, 40). However, it is important to distinguish the opposite inflammatory-regulating effects of ω-3 PUFA and ω-6 PUFA. ω-6 PUFA-derived eicosanoids were generally considered proinflammatory (41, 42). Inflammation of visceral adipose tissue leads to ectopic deposition of fat in the liver (NAFLD), which is further exacerbated by the upregulation of nuclear factor-κB (NF-κB) (43). Conversely, ω-3 PUFA can exert an anti-inflammatory effect by regulating the subunit abundance of NF-κB (44). DFP1, by increasing ω-3 PUFA, also improved the ω-6/ω-3 ratio, which was thought to reduce NAFLD risk (45, 46). Interestingly, we also found that the mediating effect of LA, one of ω-6 PUFA, was associated with a reduced risk of NAFLD, and the mediator proportion of ω-6 PUFA was smaller than that of LA. This appears to result from the risk effect of non-LA ω-6 PUFA with NAFLD (12). Several potential mechanisms could explain the observed findings. Unlike ω-6 PUFA-derived eicosanoids, LA does not increase inflammation even at high doses (47). However, LA is partially converted *in vivo* to arachidonic acid (AA), which has long been recognized as a pro-inflammatory fatty acid. Therefore, it is likely that the pro-inflammatory effects exhibited by ω-6 including AA partially offset the anti-NAFLD effect of LA. In addition, LA may reduce the risk of NAFLD by reducing ceramides, which may play a role through *de novo* hepatic lipogenesis (DNL) in diet-induced NAFLD (9, 48).

The effect of PUFA diet could be offset by the intake of red meat. DFP2 was enriched with protein, vitamins B6, B12, D, and iron, which likely represents red meat, poultry, and fish enriched diet. Red meat intake was positively associated with NAFLD risk (49, 50). As a result, the reason why PUFA-rich, DFP2 was not associated with NAFLD risk, can possibly be attributed to the offsetting effect of high meat consumption. The beneficial effects of PUFA intake included increasing ω-3 PUFA and decreasing of SFA and ω-6/ω-3 PUFA ratio. However, these effects may be masked by the deleterious effects of heme iron, resulting in an insignificant association between DFP2 and NAFLD (51). Our results suggested a diverse diet characterized by red meat intake may not increase NAFLD risk.

There were limitations in our study. First, our basis study design is a cross-sectional epidemiological study which is not suitable for causal relationship inference. The mediating effects of serum fatty acids only provide evidence for the effect of diet patterns. As such, these results should be considered preliminary. Large controlled studies are needed to confirm these findings. Second, hepatic fat, lipids, and nutrient data were not measured at a single visit, thus uncertainty exists in the interpretation of our results. Third, MUFA intake was calculated from 20 nutrients provided by UK Biobank resources for the current analysis. The MUFA intake obtained by this method incorporates the amount of total glycerol and trans fatty acids, which may result in an overestimation of MUFA intake. Therefore, it is necessary to further confirm our findings with better defined nutrients data.

In conclusion, our findings reveal that dietary fatty acids establish a crucial bridge between diet and NAFLD. The treatment of simple liver steatosis may benefit from a PUFA enriched vegetarian diet by increasing the intake of LA and DHA. “PUFA enriched carnivore” diet, representing a more common diet with high dietary adherence and food variety, is not associated with the risk of NAFLD. Our study provides valuable lifestyle guidance for the prevention and treatment of NAFLD.

## Data availability statement

The original contributions presented in the study are included in the article/Supplementary material, further inquiries can be directed to the corresponding author.

## Ethics statement

Ethics approval for the UK Biobank study was obtained from the North West Centre for Research Ethics Committee (11/NW/0382). The patients/participants provided their written informed consent to participate in this study.

## Author contributions

AT and PC designed the study. PC contributed to the acquisition of the UKB data. AT analyzed the data and wrote the first draft of the manuscript. AT and ZS performed the statistical analysis. AT, PC, ZS, MZ, JL, and XP interpreted the results. All authors revised the manuscript and approved the submission.

## Funding

This work was supported by the “Changbai Mountain Scholar” Distinguished Professor Awarding Program of the Department of Education of Jilin Province, China.

## Conflict of interest

The authors declare that the research was conducted in the absence of any commercial or financial relationships that could be construed as a potential conflict of interest.

## Publisher’s note

All claims expressed in this article are solely those of the authors and do not necessarily represent those of their affiliated organizations, or those of the publisher, the editors and the reviewers. Any product that may be evaluated in this article, or claim that may be made by its manufacturer, is not guaranteed or endorsed by the publisher.

## Footnotes

^1^ www.ceu.ox.ac.uk/research/oxford-webq

^2^ https://www.r-project.org/

## Abbreviations

NAFLD: nonalcoholic fatty liver disease
HCC: hepatocellular carcinoma
HBV: Hepatitis B virus
HCV: Hepatitis C virus
SFA: saturated fatty acid
MUFA: monounsaturated fatty acid
PUFA: polyunsaturated fatty acid
ω-3 PUFA: omega-3 PUFA
ω-6 PUFA: omega-6 PUFA
LA: linoleic Acid
AA: arachidonic acid
DHA: Docosahexaenoic Acid
PCA: principal component analysis
DFP: dietary fat intake patterns
WL-PDFF: whole liver proton density fat fraction
PDFF: proton density fat fraction
BMI: body mass index
GSH: glutathione
GGT: γ-glutamyltransferase
AST: aspartate aminotransferase
ALT: alanine aminotransferase
ALP: alkaline phosphatase
APOA: apolipoprotein A
APOB: apolipoprotein B
TC: total cholesterol
TG: triglycerides
LDL-C: low-density lipoprotein cholesterol
HDL-C: high-density lipoprotein cholesterol
