# Supplementary material for: Associations between dietary fatty acid patterns and non-alcoholic fatty liver disease in typical dietary population: A UK biobank study
Source: Front Nutr. 2023 Feb 7;10:1117626. doi: 10.3389/fnut.2023.1117626 (PMC9942598; doi:10.3389/fnut.2023.1117626)
Supplement: Supplementary file 1 [file Data_Sheet_1.docx]

# Supplementary material

## Associations between dietary fatty acid patterns and non-alcoholic fatty liver disease in typical dietary population: A UK Biobank Study

Aowen Tian^1,2^, Zewen Sun^3^, Miaoran Zhang^1,2^, Jiuling Li^1,2^, Xingchen Pan^3,4^, Peng Chen^1,2,3*^

### Contents

Covariates

Supplementary Fig. S1. Association between liver fat and serum fatty acids.

Supplementary Table S1. UKBB data field and criterion for sample exclusion.

Supplementary Table S2. Characterization of UKBB cohort of the participants of unrelated European ancestry.

Supplementary Table S3. Nutrients entered in PCA.

Supplementary Table S4. Loadings of dietary fat intake patterns (DFPs).

Supplementary Table S5. Sample characteristics according to different DFPs quartiles.

Supplementary Table S6. Observational association between DFPs with NAFLD and NAFLD related traits.

Supplementary Table S7. Observational association between DFPs with serum fatty acids

Supplementary Table S8. Observational association between DFP1 and NAFLD with serum metabolic biomarkers.

Supplementary Table S9. Fatty acid markers of DFP1 and NAFLD were jointly significantly associated.

Supplementary Table S10. Indirect effect mediated by serum fatty acids in the effect of DFPs on NAFLD.

### Covariates

We included age, gender, BMI, activity, sedentary time, income, and education as covariates for the analysis. We add up the time spent watching TV and the time spent using the computer to get sedentary time, and the sedentary values greater than the mean plus 3SD were removed as outliers. For activity, we take the natural logarithm of summed MET per week for all activities. MET per week for all activities ranged from 0 to 19278 min/week with a median of 1720 min/week and 75% of participants being active for ≤ 3279 min/week. MET was thus converted to pseudo-continuous quintile scores: 0-678 min/week (1), 678-1333 min/week (2), 1333-2202 min/week (3), 2202-3831 min/week (4), >3831 min/week (5), and the MET values higher than the mean plus 3SD were removed as outliers. For income, average total household income before tax as a pseudo-continuous variable: < £18,000 (1), £18,000-£30,999 (2), £31,000-£51,999 (3), £52 000-£100 000 (4) and > £100 000 (5). For education, we code qualifications (UKBB, category: 6138) by years of education: no qualifications (7 years), CSEs (10 years), O-levels/GCSEs (10 years), A levels/AS levels (13 years), other professional qualification (15 years), NVQ or HNC (19 years), college or university degree (20 years).

**Supplementary Fig. S1. Association between liver fat and serum fatty acids.** Radial axis is liver fat after correction for age, sex, and BMI.


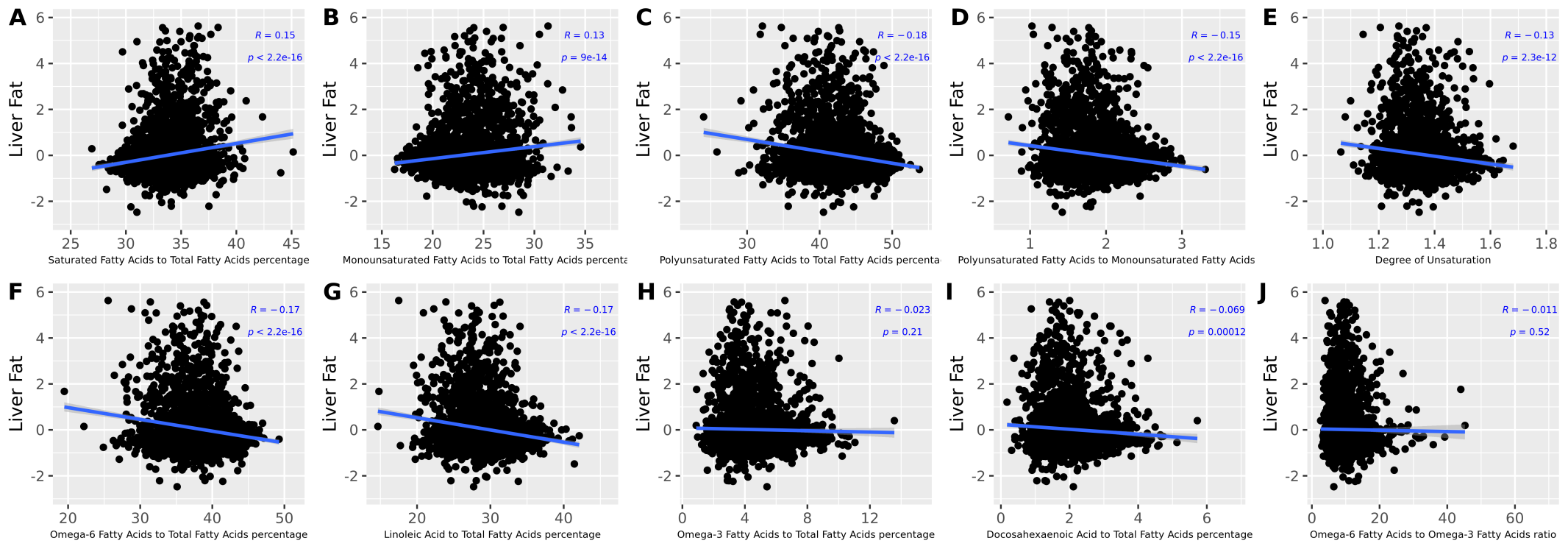


**Supplementary Table S1. UKBB data field and criterion for sample exclusion.**

| **Exclusion criteria** | **Field** | **Other information** | **sample N** |
| --- | --- | --- | --- |
| unatypical diet & unreliable energy intake | 100020&100026 | unqualified instance become NA | 20,594 |
| incomplete phenotype | 100098&21001 | NA was dropped | 3,408 |
| alcohol disease | 41270 40001 40002 20002 | ICD10:K860,K700,K701,K702,K703,K704,K709 self report:1408,1604 | 560 |
| alcohol use | 41270 40001 40002 41271 | ICD10:F101,F102 ICD9:2910,2918 | 1,778 |
| liver damage drug use | 20003 | treatment id | 291 |
|  |  | heroine:1140888836 |  |
|  |  | ammonia+ipecacuanha mixture:1140862908 |  |
|  |  | menthol+eucalyptus inhalation:1140862970 |  |
|  |  | peppermint oil product:1140865414 |  |
|  |  | foradil 12micrograms inhalation capsules+inhaler:1140923708 |  |
|  |  | virazole 6g inhalation (pdr for recon):1141165408 |  |
|  |  | ipecacuanha+morphine mixture:1140862910 |  |
|  |  | menthol+benzoin inhalation:1140862968 |  |
|  |  | menthol 35.55mg inhalant capsule:1140862870 |  |
|  |  | spiriva 18micrograms inhalation capsule: 1141182632 |  |
|  |  | virazid 6g inhalation (pdr for recon): 1140882452 |  |
|  |  | brovon inhalant: 1140881920 |  |
|  |  | rybarvin inhalant solution: 1140909398 |  |
|  |  | methyltestosterone product: 1140857656 |  |
|  |  | ethinyloestradiol+ethynodiol diacetate 30mcg/2mg tablet: 1140869172 |  |
|  |  | ethynodiol diacetate: 1140869360 |  |
| haemochromatosis | 41270 40001 40002 41271 20002 | ICD10:E831 ICD9:2750 self report:1507 | 432 |
| hbv hcv infection | 23016-23019 |  | 3,653 |
| viral hepatitis | 41270 40001 40002 41271 20002 | ICD10:B159,B160,B169,B170,B171,B172,B178,B179,B180,B181,B182,B188,B199 ICD9:0701,0703,0705,0709,5731 self report:1156,1578,1579,1580,1581,1582 | 940 |
| wernike | 41270 40001 40002 | ICD10:E512 | 0 |
| wilson | 41270 40001 40002 | ICD10:E830 | 1 |
| retain only unrelated European ancestry |  |  | 66,794 |

**Supplementary Table S2: Characterization of UKBB cohort of the participants of unrelated European ancestry**

|  | **Mean（SD）or sample size** |
| --- | --- |
| **Age** | 56.8(7.8) |
| **Gender** |  |
| Female | 49217(52.7%) |
| Male | 44182(47.3%) |
| **BMI(kg/m^2^)** | 26.9 (4.6) |
| **Income** |  |
| < £18,000 | 13,550 |
| £18,000-£30,999 | 22,951 |
| £31,000-£51,999 | 26,850 |
| £52 000-£100 000 | 234,293 |
| > £100 000 | 6,755 |
| **Education** |  |
| 7 years | 6,957 |
| 10 years | 22,833 |
| 13 years | 12,664 |
| 15 years | 4,458 |
| 19 years | 4,967 |
| 20 years | 41,520 |
| **MET(m)** | 2497.2(2399.5) |
| **Sedentary time(h)** | 3.7(1.9) |
| **WL-PDFF(%)** | 4.1(3.0) |
| **NAFLD** |  |
| Case | 2,353 |
| Control | 10,485 |
| **GGT(U/L)** | 34.4(33.9) |
| **AST(U/L)** | 25.8(9.0) |
| **ALT(U/L)** | 22.9(13.1) |
| **ALP(U/L)** | 81.1(24.8) |
| **Apolipoprotein A(g/L)** | 1.6(0.3) |
| **Apolipoprotein B(g/L)** | 1.0(0.2) |
| **TC(mmol/L)** | 5.7(1.1) |
| **TG(mmol/L)** | 1.7(1.0) |
| **LDL-C(mmol/L)** | 3.6(0.8) |
| **HDL-C(mmol/L)** | 1.5(0.4) |

*Definition of abbreviations:* BMI, Body mass index; MET, Metabolic Equivalent Task minutes; WL-PDFF, whole liver proton density fat fraction; GGT, Gamma glutamyltransferase; AST, Aspartate aminotransferase; ALT, Alanine aminotransferase; ALP, Alkaline phosphatase; TC, Total cholesterol; TG, Triglycerides; LDL-C, LDL cholesterol; HDL-C, HDL cholesterol.

**Supplementary Table S3: Nutrients entered in PCA**

| **Category** | **Description** |
| --- | --- |
| 100003 | Protein |
| 100006 | Saturated fat |
| 100007 | Polyunsaturated fat |
| 100008 | Total sugars |
| 100009 | Englyst dietary fibre |
| 100011 | Iron |
| 100012 | Vitamin B6 |
| 100013 | Vitamin B12 |
| 100014 | Folate |
| 100015 | Vitamin C |
| 100016 | Potassium |
| 100017 | Magnesium |
| 100018 | Retinol |
| 100019 | Carotene |
| 100021 | Vitamin D |
| 100023 | Starch |
| 100024 | Calcium |
| 100025 | Vitamin E |
|  | Monounsaturated fat* |

*We calculated monounsaturated fatty acid intake by subtracting saturated fatty acid (UKBB,category: 100006) and polyunsaturated fatty acid (UKBB,category: 100007) intake from total fat intake (UKBB,category: 100004).

**Supplementary Table S4: Loadings of dietary fat intake patterns (DFPs)**

|  | **DFP1** | **DFP2** | **DFP3** | **DFP4** |
| --- | --- | --- | --- | --- |
| **Proportion Var** | **0.26** | **0.13** | **0.12** | **0.12** |
| **Protein** | 0.19 | 0.68 | -0.08 | -0.1 |
| **Saturated fat** | -0.32 | -0.04 | 0.78 | 0.1 |
| **Polyunsaturated fat** | 0.1 | -0.01 | 0.02 | 0.81 |
| **Total sugars** | 0.4 | -0.19 | 0.05 | -0.62 |
| **Englyst dietary fibre** | 0.81 | -0.02 | -0.23 | 0.07 |
| **Iron** | 0.54 | 0.29 | -0.4 | 0.02 |
| **Vitamin B6** | 0.64 | 0.42 | -0.25 | -0.12 |
| **Vitamin B12** | 0.04 | 0.88 | 0.12 | 0 |
| **Folate** | 0.77 | 0.16 | -0.04 | -0.13 |
| **Vitamin C** | 0.73 | -0.02 | 0.03 | -0.21 |
| **Potassium** | 0.78 | 0.23 | -0.16 | -0.28 |
| **Magnesium** | 0.7 | 0.22 | -0.3 | -0.12 |
| **Retinol** | -0.13 | 0.01 | 0.77 | 0.11 |
| **Carotene** | 0.72 | 0.01 | 0.07 | 0.01 |
| **Vitamin D** | 0.04 | 0.77 | 0.14 | 0.19 |
| **Starch** | -0.13 | -0.23 | -0.52 | 0.2 |
| **Calcium** | 0.28 | 0.23 | 0.31 | -0.46 |
| **Vitamin E** | 0.64 | -0.12 | 0.25 | 0.45 |
| **Monounsaturated fat** | -0.17 | 0.12 | 0.53 | 0.68 |

Proportion Var,total variance each nutrient intake pattern explained.

**Supplementary Table S5: Sample characteristics according to different DFPs quartiles**

|  |  | **Mean (SD) or sample size of quartiles** | | | |  |
| --- | --- | --- | --- | --- | --- | --- |
|  |  | **1 (lowest)** | **2** | **3** | **4** | **p‐Value for trend** |
| DFP1 | **Age** | 55.6(8) | 56.9(7.9) | 57.3(7.7) | 57.4(7.6) | <0.001 |
|  | **Gender** |  |  |  |  | <0.001 |
|  | Female | 8501 | 11125 | 13306 | 16285 |  |
|  | Male | 14849 | 12225 | 10043 | 7065 |  |
|  | **BMI** | 27.6(4.7) | 26.8(4.5) | 26.5(4.4) | 26.4(4.6) | <0.001 |
|  | **WL-PDFF(%)** | 4.8(3.6) | 4.2(2.9) | 3.9(2.6) | 3.8(2.7) | <0.001 |
|  | **NAFLD** |  |  |  |  | <0.001 |
|  | Case | 775 | 606 | 539 | 433 |  |
|  | Control | 2287 | 2676 | 2784 | 2738 |  |
|  | **GGT(U/L)** | 39.7(40) | 34.9(34.4) | 32.5(30) | 30.2(29.6) | <0.001 |
|  | **AST(U/L)** | 26.3(9.2) | 25.8(9.8) | 25.7(8.9) | 25.3(8) | <0.001 |
|  | **ALT(U/L)** | 24.8(14.4) | 23.1(13) | 22.4(12.9) | 21.4(11.9) | <0.001 |
|  | **ALP(U/L)** | 81.7(27.1) | 80.7(24.3) | 80.8(23.5) | 81.3(24.2) | <0.001 |
|  | **Apolipoprotein A(g/L)** | 1.5(0.3) | 1.5(0.3) | 1.6(0.3) | 1.6(0.3) | <0.001 |
|  | **Apolipoprotein B(g/L)** | 1(0.2) | 1(0.2) | 1(0.2) | 1(0.2) | <0.001 |
|  | **TC(mmol/L)** | 5.7(1.1) | 5.7(1.1) | 5.7(1.1) | 5.7(1.1) | <0.001 |
|  | **TG(mmol/L)** | 1.8(1.1) | 1.7(1) | 1.6(0.9) | 1.6(0.9) | <0.001 |
|  | **LDL-C(mmol/L)** | 3.6(0.9) | 3.6(0.8) | 3.6(0.8) | 3.6(0.9) | 0.111 |
|  | **HDL-C(mmol/L)** | 1.4(0.4) | 1.5(0.4) | 1.5(0.4) | 1.5(0.4) | <0.001 |
|  | **Fat intake(g/1000kcal)** | 37.8(7.6) | 37.2(7.1) | 36.1(7.2) | 33.9(7.9) | <0.001 |
|  | **SFA intake(g/1000kcal)** | 15.3(4) | 14.5(3.6) | 13.6(3.4) | 12.2(3.5) | <0.001 |
|  | **PUFA intake(g/1000kcal)** | 6.2(2.4) | 6.7(2.5) | 6.9(2.6) | 7(2.8) | <0.001 |
|  | **MUFA intake(g/1000kcal)** | 16.3(3.5) | 16(3.4) | 15.6(3.5) | 14.8(4) | <0.001 |
|  | **Total energy intake (1000kcal)** | 2.2(0.6) | 2.2(0.5) | 2.1(0.5) | 1.9(0.5) | <0.001 |
| DFP2 | **Age** | 55.9(8) | 56.7(7.9) | 57.2(7.7) | 57.3(7.6) | <0.001 |
|  | **Gender** |  |  |  |  | <0.001 |
|  | Female | 11817 | 11605 | 12299 | 13496 |  |
|  | Male | 11533 | 11745 | 11050 | 9854 |  |
|  | **BMI** | 26.5(4.6) | 26.8(4.5) | 27(4.6) | 27.1(4.6) | <0.001 |
|  | **WL-PDFF(%)** | 4(2.9) | 4.2(3) | 4.1(3) | 4.2(3.1) | 0.007 |
|  | **NAFLD** |  |  |  |  | 0.7 |
|  | Case | 524 | 656 | 592 | 581 |  |
|  | Control | 2557 | 2576 | 2768 | 2584 |  |
|  | **GGT(U/L)** | 33(32.4) | 34.6(33.6) | 35.1(34.7) | 34.6(35) | <0.001 |
|  | **AST(U/L)** | 25.8(10.3) | 25.8(8.3) | 25.8(8.3) | 25.8(8.9) | 0.936 |
|  | **ALT(U/L)** | 22.5(13.4) | 23(12.9) | 23.2(12.9) | 23(13.3) | <0.001 |
|  | **ALP(U/L)** | 81.3(25.1) | 81.3(25.5) | 81.2(24.8) | 80.7(23.9) | 0.012 |
|  | **Apolipoprotein A(g/L)** | 1.5(0.3) | 1.5(0.3) | 1.6(0.3) | 1.6(0.3) | <0.001 |
|  | **Apolipoprotein B(g/L)** | 1(0.2) | 1(0.2) | 1(0.2) | 1(0.2) | <0.001 |
|  | **TC(mmol/L)** | 5.7(1.1) | 5.7(1.1) | 5.7(1.1) | 5.7(1.1) | <0.001 |
|  | **TG(mmol/L)** | 1.7(1) | 1.7(1) | 1.7(1) | 1.6(1) | <0.001 |
|  | **LDL-C(mmol/L)** | 3.5(0.8) | 3.6(0.9) | 3.6(0.9) | 3.6(0.9) | 0.005 |
|  | **HDL-C(mmol/L)** | 1.5(0.4) | 1.5(0.4) | 1.5(0.4) | 1.5(0.4) | <0.001 |
|  | **Fat intake(g/1000kcal)** | 36.2(7.7) | 36.3(7.2) | 36.1(7.4) | 36.4(8) | <0.001 |
|  | **SFA intake(g/1000kcal)** | 14(3.9) | 14.1(3.7) | 14(3.7) | 13.6(3.9) | <0.001 |
|  | **PUFA intake(g/1000kcal)** | 6.9(2.8) | 6.7(2.5) | 6.5(2.4) | 6.6(2.5) | <0.001 |
|  | **MUFA intake(g/1000kcal)** | 15.3(3.7) | 15.5(3.4) | 15.6(3.5) | 16.2(4) | <0.001 |
|  | **Total energy intake (1000kcal)** | 2.2(0.6) | 2.2(0.5) | 2(0.5) | 1.9(0.5) | <0.001 |
| DFP3 | **Age** | 56.4(7.8) | 56.8(7.8) | 57(7.9) | 57.1(7.8) | <0.001 |
|  | **Gender** |  |  |  |  | <0.001 |
|  | Female | 10647 | 11820 | 12733 | 14017 |  |
|  | Male | 12703 | 11530 | 10616 | 9333 |  |
|  | **BMI** | 26.9(4.4) | 26.8(4.5) | 26.7(4.6) | 26.9(4.8) | <0.001 |
|  | **WL-PDFF(%)** | 4.2(3) | 4.2(3) | 4.1(3) | 4.1(3) | 0.22 |
|  | **NAFLD** |  |  |  |  | 0.517 |
|  | Case | 579 | 671 | 541 | 562 |  |
|  | Control | 2583 | 2768 | 2675 | 2459 |  |
|  | **GGT(U/L)** | 36.3(35.1) | 34.9(34.1) | 33.3(33.2) | 32.8(33.2) | <0.001 |
|  | **AST(U/L)** | 26.2(8.8) | 26(9) | 25.6(9.8) | 25.4(8.4) | <0.001 |
|  | **ALT(U/L)** | 23.6(13.2) | 23.2(12.9) | 22.5(13.2) | 22.4(13.2) | <0.001 |
|  | **ALP(U/L)** | 80.5(24.5) | 81.1(24.5) | 81.1(24) | 81.9(26.3) | <0.001 |
|  | **Apolipoprotein A(g/L)** | 1.5(0.3) | 1.5(0.3) | 1.6(0.3) | 1.6(0.3) | <0.001 |
|  | **Apolipoprotein B(g/L)** | 1(0.2) | 1(0.2) | 1(0.2) | 1(0.2) | <0.001 |
|  | **TC(mmol/L)** | 5.6(1.1) | 5.7(1.1) | 5.7(1.1) | 5.8(1.1) | <0.001 |
|  | **TG(mmol/L)** | 1.7(1) | 1.7(1) | 1.7(1) | 1.7(1) | <0.001 |
|  | **LDL-C(mmol/L)** | 3.5(0.9) | 3.6(0.8) | 3.6(0.8) | 3.6(0.9) | <0.001 |
|  | **HDL-C(mmol/L)** | 1.5(0.4) | 1.5(0.4) | 1.5(0.4) | 1.5(0.4) | <0.001 |
|  | **Fat intake(g/1000kcal)** | 30(6.5) | 34.8(5.7) | 37.8(5.7) | 42.4(6.5) | <0.001 |
|  | **SFA intake(g/1000kcal)** | 10.2(2.5) | 12.9(2.3) | 14.7(2.4) | 17.8(3.2) | <0.001 |
|  | **PUFA intake(g/1000kcal)** | 6.6(2.9) | 6.7(2.5) | 6.7(2.4) | 6.7(2.5) | <0.001 |
|  | **MUFA intake(g/1000kcal)** | 13.2(3.1) | 15.1(3) | 16.3(3.1) | 18(3.6) | <0.001 |
|  | **Total energy intake (1000kcal)** | 2(0.5) | 2.1(0.5) | 2.1(0.5) | 2.1(0.6) | <0.001 |
| DFP4 | **Age** | 57.3(7.7) | 57.2(7.8) | 56.7(7.8) | 56(8) | <0.001 |
|  | **Gender** |  |  |  |  | 0.035 |
|  | Female | 12754 | 11950 | 12024 | 12489 |  |
|  | Male | 10596 | 11400 | 11325 | 10861 |  |
|  | **BMI** | 27(4.5) | 26.8(4.5) | 26.7(4.6) | 26.8(4.8) | <0.001 |
|  | **WL-PDFF(%)** | 4.2(3.1) | 4.1(3) | 4(2.9) | 4.2(3.1) | 0.119 |
|  | **NAFLD** |  |  |  |  | 0.439 |
|  | Case | 573 | 604 | 595 | 581 |  |
|  | Control | 2452 | 2689 | 2756 | 2588 |  |
|  | **GGT(U/L)** | 35.2(36.6) | 34.7(34) | 34.3(33.6) | 33.2(31.4) | <0.001 |
|  | **AST(U/L)** | 26(10.1) | 25.8(8.3) | 25.7(8.3) | 25.6(9.2) | <0.001 |
|  | **ALT(U/L)** | 23.1(14) | 22.9(12.5) | 22.9(12.8) | 22.8(13.1) | 0.123 |
|  | **ALP(U/L)** | 81.9(28.1) | 81(23.3) | 80.7(23.3) | 80.9(24.3) | <0.001 |
|  | **Apolipoprotein A(g/L)** | 1.5(0.3) | 1.6(0.3) | 1.6(0.3) | 1.6(0.3) | 0.008 |
|  | **Apolipoprotein B(g/L)** | 1(0.2) | 1(0.2) | 1(0.2) | 1(0.2) | <0.001 |
|  | **TC(mmol/L)** | 5.7(1.1) | 5.7(1.1) | 5.7(1.1) | 5.7(1.1) | <0.001 |
|  | **TG(mmol/L)** | 1.7(1) | 1.7(1) | 1.7(1) | 1.7(1) | 0.001 |
|  | **LDL-C(mmol/L)** | 3.6(0.8) | 3.6(0.8) | 3.6(0.8) | 3.5(0.9) | <0.001 |
|  | **HDL-C(mmol/L)** | 1.5(0.4) | 1.5(0.4) | 1.5(0.4) | 1.5(0.4) | 0.098 |
|  | **Fat intake(g/1000kcal)** | 30(6.4) | 34.6(5.8) | 37.8(5.6) | 42.6(6.4) | <0.001 |
|  | **SFA intake(g/1000kcal)** | 13.2(4) | 14(3.8) | 14.3(3.7) | 14.2(3.6) | <0.001 |
|  | **PUFA intake(g/1000kcal)** | 4.3(1.4) | 5.8(1.4) | 7.1(1.5) | 9.6(2.3) | <0.001 |
|  | **MUFA intake(g/1000kcal)** | 12.6(2.8) | 14.9(2.5) | 16.4(2.6) | 18.8(3.4) | <0.001 |
|  | **Total energy intake (1000kcal)** | 1.9(0.5) | 2.1(0.5) | 2.1(0.5) | 2.2(0.6) | <0.001 |

*Definition of abbreviations:*BMI, Body mass index; WL-PDFF, whole liver proton density fat fraction; GGT, Gamma glutamyltransferase; AST, Aspartate aminotransferase; ALT, Alanine aminotransferase; ALP, Alkaline phosphatase; TC, Total cholesterol; TG, Triglycerides; LDL-C, LDL cholesterol; HDL-C, HDL cholesterol; SFA, Saturated fatty acids; PUFA, Polyunsaturated fatty acids; MUFA, Monounsaturated fatty acids.

**Supplementary Table S6: Observational association between DFPs with NAFLD and NAFLD related traits**

**A. Observational association between DFPs and NAFLD**

| **Model** | **DFPs** | **n** | **OR** | **CI Lower 95%** | **CI Upper 95%** | **FDR p** |
| --- | --- | --- | --- | --- | --- | --- |
| **Minimum-adjustment model^1^** | DFP1 | 12,838 | 0.82 | 0.78 | 0.87 | 2.92E-11 |
|  | DFP2 | 12,838 | 0.99 | 0.94 | 1.04 | 0.58 |
|  | DFP3 | 12,838 | 1.03 | 0.98 | 1.08 | 0.45 |
|  | DFP4 | 12,838 | 0.98 | 0.93 | 1.03 | 0.45 |
| **Fully-adjusted model^2^** | DFP1 | 10,623 | 0.83 | 0.78 | 0.88 | 1.11E-08 |
|  | DFP2 | 10,623 | 0.99 | 0.94 | 1.05 | 0.70 |
|  | DFP3 | 10,623 | 1.03 | 0.97 | 1.09 | 0.39 |
|  | DFP4 | 10,623 | 0.97 | 0.91 | 1.02 | 0.39 |

**B. Observational association between DFP1 and NAFLD related traits**

| **Model** | **Traits** | **n** | **beta** | **CI Lower 95%** | **CI Upper 95%** | **FDR p** |
| --- | --- | --- | --- | --- | --- | --- |
| **Minimum-adjustment model^1^** | Liver Fat | 12,838 | -0.22 | -0.27 | -0.17 | 3.67E-16 |
|  | BMI | 93,399 | -0.30 | -0.33 | -0.27 | 2.01E-81 |
|  | GGT | 88,922 | -1.64 | -1.86 | -1.42 | 2.18E-46 |
|  | AST | 88,661 | 0.10 | 0.04 | 0.16 | 6.93E-04 |
|  | ALT | 88,927 | -0.11 | -0.19 | -0.03 | 0.01 |
|  | ALP | 88,969 | -0.58 | -0.74 | -0.41 | 8.68E-12 |
|  | Apolipoprotein A | 81,219 | -0.01 | -0.01 | -0.01 | 1.05E-22 |
|  | Apolipoprotein B | 88,575 | -0.01 | -0.01 | 0.00 | 6.06E-15 |
|  | Cholesterol | 88,957 | -0.04 | -0.05 | -0.03 | 2.19E-25 |
|  | TC | 88,899 | -0.03 | -0.03 | -0.02 | 6.12E-18 |
|  | LDL-C | 88,797 | -0.03 | -0.03 | -0.02 | 1.15E-18 |
|  | HDL-C | 81,665 | 0.00 | -0.01 | 0.00 | 1.06E-04 |
| **Fully-adjusted model^2^** | Liver Fat | 10,623 | -0.21 | -0.27 | -0.15 | 3.29E-12 |
|  | BMI | 75,692 | -0.12 | -0.15 | -0.09 | 5.92E-13 |
|  | GGT | 72,047 | -1.46 | -1.71 | -1.21 | 6.90E-30 |
|  | AST | 71,835 | 0.09 | 0.03 | 0.16 | 4.78E-03 |
|  | ALT | 72,049 | -0.02 | -0.12 | 0.07 | 0.65 |
|  | ALP | 72,084 | -0.30 | -0.49 | -0.12 | 1.70E-03 |
|  | Apolipoprotein A | 65,835 | -0.01 | -0.01 | -0.01 | 6.41E-34 |
|  | Apolipoprotein B | 71,766 | -0.01 | -0.01 | 0.00 | 4.83E-11 |
|  | Cholesterol | 72,075 | -0.05 | -0.05 | -0.04 | 6.29E-26 |
|  | TC | 72,032 | -0.02 | -0.03 | -0.01 | 3.83E-07 |
|  | LDL-C | 71,948 | -0.03 | -0.03 | -0.02 | 3.75E-17 |
|  | HDL-C | 66,196 | -0.01 | -0.01 | -0.01 | 8.13E-15 |

*Definition of abbreviations:* n, the number of participants used in observational test; beta, effect size; OR, odds ratio; CI Lower 95%, lower bound of the 95% confidence interval; CI Upper 95%, upper limit of the 95% confidence interval; FDR p, p value after FDR corrected.

^1^Minimum-adjustment model: adjusted for age, gender, and BMI.

^2^Fully-adjusted model: adjusted for age, gender, BMI, sedentary time, exercise, income, and education.

**Supplementary Table S7: Observational association between DFPs with serum fatty acids**

|  | **Description** | **n** | **beta** | **CI Lower 95%** | **CI Upper 95%** | **FDR p** |
| --- | --- | --- | --- | --- | --- | --- |
| DFP1 | Omega-3 Fatty Acids to Total Fatty Acids percentage | 22,194 | 0.15 | 0.13 | 0.17 | 3.00E-46 |
|  | Omega-6 Fatty Acids to Total Fatty Acids percentage | 22,194 | 0.22 | 0.18 | 0.27 | 1.98E-22 |
|  | Polyunsaturated Fatty Acids to Total Fatty Acids percentage | 22,194 | 0.37 | 0.33 | 0.42 | 2.31E-57 |
|  | Monounsaturated Fatty Acids to Total Fatty Acids percentage | 22,194 | -0.14 | -0.17 | -0.11 | 2.10E-18 |
|  | Saturated Fatty Acids to Total Fatty Acids percentage | 22,194 | -0.23 | -0.25 | -0.20 | 2.90E-67 |
|  | Linoleic Acid to Total Fatty Acids percentage | 22,194 | 0.19 | 0.15 | 0.23 | 1.91E-18 |
|  | Docosahexaenoic Acid to Total Fatty Acids percentage | 22,194 | 0.06 | 0.06 | 0.07 | 2.42E-47 |
|  | Polyunsaturated Fatty Acids to Monounsaturated Fatty Acids ratio | 22,194 | 0.03 | 0.02 | 0.03 | 1.15E-36 |
|  | Omega-6 Fatty Acids to Omega-3 Fatty Acids ratio | 22,194 | -0.23 | -0.28 | -0.18 | 4.73E-17 |
| DFP2 | Omega-3 Fatty Acids to Total Fatty Acids percentage | 22,194 | 0.32 | 0.30 | 0.34 | 2.11E-228 |
|  | Omega-6 Fatty Acids to Total Fatty Acids percentage | 22,194 | -0.06 | -0.10 | -0.02 | 8.18E-03 |
|  | Polyunsaturated Fatty Acids to Total Fatty Acids percentage | 22,194 | 0.26 | 0.22 | 0.31 | 8.17E-32 |
|  | Monounsaturated Fatty Acids to Total Fatty Acids percentage | 22,194 | -0.26 | -0.29 | -0.23 | 1.42E-59 |
|  | Saturated Fatty Acids to Total Fatty Acids percentage | 22,194 | -0.01 | -0.03 | 0.02 | 0.57 |
|  | Linoleic Acid to Total Fatty Acids percentage | 22,194 | -0.24 | -0.29 | -0.20 | 8.71E-32 |
|  | Docosahexaenoic Acid to Total Fatty Acids percentage | 22,194 | 0.13 | 0.13 | 0.14 | 1.12E-221 |
|  | Polyunsaturated Fatty Acids to Monounsaturated Fatty Acids ratio | 22,194 | 0.03 | 0.03 | 0.04 | 2.33E-57 |
|  | Omega-6 Fatty Acids to Omega-3 Fatty Acids ratio | 22,194 | -0.71 | -0.76 | -0.66 | 8.01E-167 |
| DFP3 | Omega-3 Fatty Acids to Total Fatty Acids percentage | 22,194 | -0.10 | -0.12 | -0.08 | 7.34E-25 |
|  | Omega-6 Fatty Acids to Total Fatty Acids percentage | 22,194 | 0.01 | -0.03 | 0.05 | 0.72 |
|  | Polyunsaturated Fatty Acids to Total Fatty Acids percentage | 22,194 | -0.10 | -0.14 | -0.05 | 2.34E-05 |
|  | Monounsaturated Fatty Acids to Total Fatty Acids percentage | 22,194 | 0.00 | -0.03 | 0.03 | 0.92 |
|  | Saturated Fatty Acids to Total Fatty Acids percentage | 22,194 | 0.09 | 0.07 | 0.12 | 1.34E-13 |
|  | Linoleic Acid to Total Fatty Acids percentage | 22,194 | 0.09 | 0.05 | 0.13 | 1.27E-05 |
|  | Docosahexaenoic Acid to Total Fatty Acids percentage | 22,194 | -0.04 | -0.05 | -0.03 | 5.80E-19 |
|  | Polyunsaturated Fatty Acids to Monounsaturated Fatty Acids ratio | 22,194 | 0.00 | -0.01 | 0.00 | 0.08 |
|  | Omega-6 Fatty Acids to Omega-3 Fatty Acids ratio | 22,194 | 0.25 | 0.20 | 0.30 | 1.23E-21 |
| DFP4 | Omega-3 Fatty Acids to Total Fatty Acids percentage | 22,194 | -0.03 | -0.05 | -0.01 | 1.50E-03 |
|  | Omega-6 Fatty Acids to Total Fatty Acids percentage | 22,194 | 0.29 | 0.25 | 0.34 | 9.23E-42 |
|  | Polyunsaturated Fatty Acids to Total Fatty Acids percentage | 22,194 | 0.26 | 0.22 | 0.30 | 9.27E-32 |
|  | Monounsaturated Fatty Acids to Total Fatty Acids percentage | 22,194 | 0.03 | 0.00 | 0.06 | 0.08 |
|  | Saturated Fatty Acids to Total Fatty Acids percentage | 22,194 | -0.29 | -0.31 | -0.26 | 1.24E-117 |
|  | Linoleic Acid to Total Fatty Acids percentage | 22,194 | 0.41 | 0.37 | 0.45 | 1.59E-89 |
|  | Docosahexaenoic Acid to Total Fatty Acids percentage | 22,194 | -0.01 | -0.01 | 0.00 | 0.25 |
|  | Polyunsaturated Fatty Acids to Monounsaturated Fatty Acids ratio | 22,194 | 0.01 | 0.00 | 0.01 | 2.32E-04 |
|  | Omega-6 Fatty Acids to Omega-3 Fatty Acids ratio | 22,194 | 0.15 | 0.10 | 0.20 | 1.45E-08 |

*Definition of abbreviations:* n, the number of participants used in observational test; beta, effect size; OR, odds ratio; CI Lower 95%, lower bound of the 95% confidence interval; CI Upper 95%, upper limit of the 95% confidence interval; FDR p, p value after FDR corrected.

**Supplementary Table S8: Observational association between DFP1 and NAFLD with serum metabolic biomarkers**

| **Serum metabolic biomarkers** | | **DFP1** | | | | | **NAFLD** | | | | |
| --- | --- | --- | --- | --- | --- | --- | --- | --- | --- | --- | --- |
| **Group** | **Description** | **n** | **beta** | **CI Lower 95%** | **CI Upper 95%** | **FDR p** | **n** | **OR** | **CI Lower 95%** | **CI Upper 95%** | **FDR p** |
| Cholesterol | Total Cholesterol | 22,194 | -0.02 | -0.04 | -0.01 | 9.94E-04 | 3,087 | 1.03 | 0.94 | 1.14 | 5.65E-01 |
| Cholesterol | Total Cholesterol Minus HDL-C | 22,194 | -0.02 | -0.04 | -0.01 | 1.55E-03 | 3,087 | 1.12 | 1.01 | 1.23 | 3.49E-02 |
| Cholesterol | Remnant Cholesterol (Non-HDL, Non-LDL -Cholesterol) | 22,194 | -0.02 | -0.04 | -0.01 | 2.42E-03 | 3,087 | 1.14 | 1.03 | 1.25 | 1.44E-02 |
| Cholesterol | Clinical LDL Cholesterol | 22,194 | -0.02 | -0.04 | -0.01 | 2.60E-03 | 3,087 | 1.06 | 0.96 | 1.17 | 2.68E-01 |
| Cholesterol | LDL Cholesterol | 22,194 | -0.02 | -0.04 | -0.01 | 1.47E-03 | 3,087 | 1.09 | 0.99 | 1.20 | 9.14E-02 |
| Cholesterol | HDL Cholesterol | 22,194 | -0.01 | -0.02 | 0.00 | 1.37E-01 | 3,087 | 0.75 | 0.66 | 0.84 | 2.87E-06 |
| Triglycerides | Total Triglycerides | 22,194 | -0.03 | -0.05 | -0.02 | 2.03E-06 | 3,087 | 1.54 | 1.38 | 1.71 | 6.46E-14 |
| Triglycerides | Triglycerides in VLDL | 22,194 | -0.03 | -0.04 | -0.01 | 5.50E-05 | 3,087 | 1.54 | 1.38 | 1.72 | 8.94E-14 |
| Triglycerides | Triglycerides in LDL | 22,194 | -0.04 | -0.05 | -0.03 | 1.17E-08 | 3,087 | 1.43 | 1.30 | 1.59 | 1.27E-11 |
| Triglycerides | Triglycerides in HDL | 22,194 | -0.05 | -0.06 | -0.04 | 1.04E-11 | 3,087 | 1.36 | 1.23 | 1.51 | 6.48E-09 |
| Phospholipids | Total Phospholipids in Lipoprotein Particles | 22,194 | -0.03 | -0.05 | -0.02 | 9.43E-07 | 3,087 | 1.12 | 1.01 | 1.24 | 4.67E-02 |
| Phospholipids | Phospholipids in VLDL | 22,194 | -0.02 | -0.03 | -0.01 | 2.10E-03 | 3,087 | 1.39 | 1.26 | 1.54 | 8.46E-10 |
| Phospholipids | Phospholipids in LDL | 22,194 | -0.03 | -0.04 | -0.01 | 4.90E-04 | 3,087 | 1.11 | 1.01 | 1.23 | 3.96E-02 |
| Phospholipids | Phospholipids in HDL | 22,194 | -0.02 | -0.04 | -0.01 | 2.14E-04 | 3,087 | 0.89 | 0.80 | 1.00 | 6.75E-02 |
| Cholesteryl esters | Total Esterified Cholesterol | 22,194 | -0.02 | -0.04 | -0.01 | 5.88E-04 | 3,087 | 1.02 | 0.92 | 1.12 | 7.74E-01 |
| Cholesteryl esters | Cholesteryl Esters in VLDL | 22,194 | -0.01 | -0.03 | 0.00 | 5.39E-02 | 3,087 | 1.19 | 1.08 | 1.32 | 7.09E-04 |
| Cholesteryl esters | Cholesteryl Esters in LDL | 22,194 | -0.02 | -0.04 | -0.01 | 8.70E-04 | 3,087 | 1.13 | 1.02 | 1.24 | 2.20E-02 |
| Cholesteryl esters | Cholesteryl Esters in HDL | 22,194 | -0.01 | -0.02 | 0.00 | 2.09E-01 | 3,087 | 0.73 | 0.65 | 0.82 | 5.11E-07 |
| Free cholesterol | Total Free Cholesterol | 22,194 | -0.02 | -0.03 | -0.01 | 3.68E-03 | 3,087 | 1.07 | 0.97 | 1.19 | 1.90E-01 |
| Free cholesterol | Free Cholesterol in VLDL | 22,194 | -0.02 | -0.03 | -0.01 | 6.16E-03 | 3,087 | 1.35 | 1.22 | 1.49 | 2.20E-08 |
| Free cholesterol | Free Cholesterol in LDL | 22,194 | -0.02 | -0.03 | -0.01 | 7.21E-03 | 3,087 | 1.01 | 0.91 | 1.11 | 9.26E-01 |
| Free cholesterol | Free Cholesterol in HDL | 22,194 | -0.01 | -0.03 | 0.00 | 2.55E-02 | 3,087 | 0.81 | 0.72 | 0.92 | 9.83E-04 |
| Total lipids | Total Lipids in Lipoprotein Particles | 22,194 | -0.03 | -0.05 | -0.02 | 2.43E-06 | 3,087 | 1.20 | 1.09 | 1.32 | 4.78E-04 |
| Total lipids | Total Lipids in VLDL | 22,194 | -0.02 | -0.04 | -0.01 | 3.84E-04 | 3,087 | 1.45 | 1.31 | 1.61 | 1.27E-11 |
| Total lipids | Total Lipids in LDL | 22,194 | -0.03 | -0.04 | -0.01 | 4.82E-04 | 3,087 | 1.12 | 1.02 | 1.24 | 2.85E-02 |
| Total lipids | Total Lipids in HDL | 22,194 | -0.02 | -0.03 | -0.01 | 1.73E-03 | 3,087 | 0.85 | 0.76 | 0.95 | 7.01E-03 |
| Lipoprotein particle concentrations | Total Concentration of Lipoprotein Particles | 22,194 | -0.04 | -0.05 | -0.03 | 8.74E-09 | 3,087 | 0.99 | 0.89 | 1.10 | 9.13E-01 |
| Lipoprotein particle concentrations | Concentration of VLDL Particles | 22,194 | -0.02 | -0.03 | -0.01 | 6.16E-03 | 3,087 | 1.32 | 1.19 | 1.46 | 1.96E-07 |
| Lipoprotein particle concentrations | Concentration of LDL Particles | 22,194 | -0.01 | -0.02 | 0.00 | 1.37E-01 | 3,087 | 1.13 | 1.02 | 1.24 | 2.27E-02 |
| Lipoprotein particle concentrations | Concentration of HDL Particles | 22,194 | -0.04 | -0.05 | -0.03 | 1.30E-08 | 3,087 | 0.97 | 0.87 | 1.07 | 5.81E-01 |
| Lipoprotein particle sizes | Average Diameter for VLDL Particles | 22,194 | -0.02 | -0.04 | -0.01 | 2.38E-04 | 3,087 | 1.56 | 1.39 | 1.75 | 2.66E-13 |
| Lipoprotein particle sizes | Average Diameter for LDL Particles | 22,194 | 0.04 | 0.02 | 0.05 | 1.70E-07 | 3,087 | 0.71 | 0.64 | 0.78 | 1.02E-10 |
| Lipoprotein particle sizes | Average Diameter for HDL Particles | 22,194 | 0.02 | 0.01 | 0.03 | 1.61E-03 | 3,087 | 0.65 | 0.57 | 0.73 | 1.27E-11 |
| Other lipids | Phosphoglycerides | 22,194 | -0.03 | -0.05 | -0.02 | 1.83E-06 | 3,087 | 1.12 | 1.01 | 1.24 | 4.36E-02 |
| Other lipids | Triglycerides to Phosphoglycerides ratio | 22,194 | -0.02 | -0.01 | 0.00 | 1.69E-03 | 3,087 | 1.60 | 4.53 | 11.61 | 3.16E-14 |
| Other lipids | Total Cholines | 22,194 | -0.03 | -0.04 | -0.02 | 1.57E-05 | 3,087 | 1.07 | 0.97 | 1.19 | 2.29E-01 |
| Other lipids | Phosphatidylcholines | 22,194 | -0.03 | -0.05 | -0.02 | 1.65E-06 | 3,087 | 1.07 | 0.96 | 1.19 | 2.47E-01 |
| Other lipids | Sphingomyelins | 22,194 | -0.03 | -0.04 | -0.02 | 1.69E-05 | 3,087 | 1.00 | 0.90 | 1.11 | 9.80E-01 |
| Apolipoproteins | Apolipoprotein B | 22,194 | -0.01 | -0.03 | 0.00 | 5.10E-02 | 3,087 | 1.14 | 1.03 | 1.25 | 1.34E-02 |
| Apolipoproteins | Apolipoprotein A1 | 22,194 | -0.03 | -0.04 | -0.02 | 2.73E-06 | 3,087 | 0.91 | 0.82 | 1.02 | 1.22E-01 |
| Apolipoproteins | Apolipoprotein B to Apolipoprotein A1 ratio | 22,194 | 0.01 | 0.00 | 0.00 | 4.63E-01 | 3,087 | 1.18 | 1.47 | 4.80 | 2.48E-03 |
| Fatty acids | Total Fatty Acids | 22,194 | -0.03 | -0.05 | -0.02 | 4.95E-06 | 3,087 | 1.33 | 1.21 | 1.47 | 3.22E-08 |
| Fatty acids | Degree of Unsaturation | 22,194 | 0.11 | 0.01 | 0.01 | 1.07E-69 | 3,087 | 0.67 | 0.00 | 0.02 | 1.88E-12 |
| Fatty acids | Omega-3 Fatty Acids | 22,194 | 0.06 | 0.05 | 0.08 | 3.70E-19 | 3,087 | 1.08 | 0.98 | 1.19 | 1.67E-01 |
| Fatty acids | Omega-6 Fatty Acids | 22,194 | 0.00 | -0.02 | 0.01 | 6.16E-01 | 3,087 | 1.08 | 0.98 | 1.19 | 1.57E-01 |
| Fatty acids | Polyunsaturated Fatty Acids | 22,194 | 0.02 | 0.00 | 0.03 | 3.80E-02 | 3,087 | 1.09 | 0.99 | 1.20 | 1.08E-01 |
| Fatty acids | Monounsaturated Fatty Acids | 22,194 | -0.05 | -0.06 | -0.03 | 3.03E-11 | 3,087 | 1.45 | 1.31 | 1.60 | 6.70E-12 |
| Fatty acids | Saturated Fatty Acids | 22,194 | -0.06 | -0.07 | -0.05 | 4.12E-18 | 3,087 | 1.43 | 1.30 | 1.59 | 8.44E-12 |
| Fatty acids | Linoleic Acid | 22,194 | 0.00 | -0.01 | 0.01 | 9.53E-01 | 3,087 | 1.03 | 0.93 | 1.13 | 6.11E-01 |
| Fatty acids | Docosahexaenoic Acid | 22,194 | 0.07 | 0.06 | 0.08 | 6.00E-24 | 3,087 | 0.93 | 0.84 | 1.03 | 2.07E-01 |
| Fatty acids | Omega-3 Fatty Acids to Total Fatty Acids percentage | 22,194 | 0.15 | 0.13 | 0.17 | 2.92E-42 | 3,087 | 0.95 | 0.89 | 1.01 | 1.15E-01 |
| Fatty acids | Omega-6 Fatty Acids to Total Fatty Acids percentage | 22,194 | 0.22 | 0.18 | 0.27 | 5.80E-25 | 3,087 | 0.86 | 0.84 | 0.89 | 2.65E-20 |
| Fatty acids | Polyunsaturated Fatty Acids to Total Fatty Acids percentage | 22,194 | 0.37 | 0.33 | 0.42 | 1.40E-65 | 3,087 | 0.86 | 0.84 | 0.89 | 2.14E-21 |
| Fatty acids | Monounsaturated Fatty Acids to Total Fatty Acids percentage | 22,194 | -0.14 | -0.17 | -0.11 | 3.52E-17 | 3,087 | 1.18 | 1.13 | 1.23 | 3.17E-14 |
| Fatty acids | Saturated Fatty Acids to Total Fatty Acids percentage | 22,194 | -0.23 | -0.25 | -0.20 | 1.74E-71 | 3,087 | 1.24 | 1.18 | 1.30 | 1.19E-14 |
| Fatty acids | Linoleic Acid to Total Fatty Acids percentage | 22,194 | 0.19 | 0.15 | 0.23 | 1.56E-18 | 3,087 | 0.86 | 0.83 | 0.89 | 2.54E-18 |
| Fatty acids | Docosahexaenoic Acid to Total Fatty Acids percentage | 22,194 | 0.06 | 0.06 | 0.07 | 2.89E-43 | 3,087 | 0.70 | 0.59 | 0.82 | 4.04E-06 |
| Fatty acids | Polyunsaturated Fatty Acids to Monounsaturated Fatty Acids ratio | 22,194 | 0.08 | 0.02 | 0.03 | 5.30E-35 | 3,087 | 0.58 | 0.15 | 0.29 | 1.63E-18 |
| Fatty acids | Omega-6 Fatty Acids to Omega-3 Fatty Acids ratio | 22,194 | -0.07 | -0.28 | -0.18 | 1.40E-24 | 3,087 | 0.95 | 0.97 | 1.02 | 3.65E-01 |
| Amino acids | Alanine | 22,194 | 0.00 | -0.01 | 0.01 | 9.49E-01 | 3,087 | 1.07 | 0.97 | 1.18 | 2.22E-01 |
| Amino acids | Glutamine | 22,194 | 0.01 | 0.00 | 0.02 | 1.28E-01 | 3,087 | 0.93 | 0.84 | 1.02 | 1.43E-01 |
| Amino acids | Glycine | 22,194 | 0.01 | 0.00 | 0.03 | 4.29E-02 | 3,087 | 0.75 | 0.67 | 0.83 | 1.43E-07 |
| Amino acids | Histidine | 22,194 | 0.01 | 0.00 | 0.03 | 1.03E-01 | 3,087 | 1.03 | 0.93 | 1.13 | 6.48E-01 |
| Amino acids | Total Concentration of Branched-Chain Amino Acids (Leucine + Isoleucine + Valine) | 22,194 | 0.00 | -0.02 | 0.01 | 5.08E-01 | 3,087 | 1.28 | 1.15 | 1.43 | 8.78E-06 |
| Amino acids | Isoleucine | 22,194 | -0.01 | -0.02 | 0.00 | 1.03E-01 | 3,087 | 1.21 | 1.09 | 1.34 | 5.05E-04 |
| Amino acids | Leucine | 22,194 | -0.02 | -0.03 | 0.00 | 2.42E-02 | 3,087 | 1.23 | 1.11 | 1.37 | 1.98E-04 |
| Amino acids | Phenylalanine | 22,194 | -0.01 | -0.02 | 0.00 | 2.09E-01 | 3,087 | 1.08 | 0.98 | 1.18 | 1.71E-01 |
| Amino acids | Tyrosine | 22,194 | -0.01 | -0.03 | 0.00 | 5.33E-02 | 3,087 | 1.19 | 1.08 | 1.31 | 7.76E-04 |
| Glycolysis related metabolites | Glucose | 22,194 | 0.02 | 0.01 | 0.04 | 1.68E-03 | 3,087 | 1.13 | 1.02 | 1.24 | 2.54E-02 |
| Glycolysis related metabolites | Lactate | 22,194 | -0.03 | -0.04 | -0.02 | 2.51E-05 | 3,087 | 1.09 | 0.98 | 1.20 | 1.35E-01 |
| Glycolysis related metabolites | Pyruvate | 22,194 | -0.02 | -0.03 | 0.00 | 2.77E-02 | 3,087 | 1.03 | 0.93 | 1.14 | 6.35E-01 |
| Glycolysis related metabolites | Citrate | 22,194 | 0.01 | 0.00 | 0.02 | 1.28E-01 | 3,087 | 1.04 | 0.94 | 1.14 | 5.26E-01 |
| Ketone bodies | 3-Hydroxybutyrate | 22,194 | 0.01 | 0.00 | 0.03 | 7.02E-02 | 3,087 | 1.15 | 1.04 | 1.27 | 1.06E-02 |
| Ketone bodies | Acetate | 22,194 | 0.06 | 0.04 | 0.07 | 6.52E-16 | 3,087 | 0.84 | 0.76 | 0.92 | 4.74E-04 |
| Ketone bodies | Acetoacetate | 22,194 | 0.02 | 0.01 | 0.04 | 1.50E-03 | 3,087 | 1.14 | 1.03 | 1.25 | 1.27E-02 |
| Ketone bodies | Acetone | 22,194 | 0.03 | 0.01 | 0.04 | 1.81E-04 | 3,087 | 0.97 | 0.88 | 1.07 | 5.97E-01 |
| Fluid balance | Creatinine | 22,194 | -0.05 | -0.06 | -0.04 | 5.17E-15 | 3,087 | 1.16 | 1.02 | 1.31 | 3.28E-02 |
| Fluid balance | Albumin | 22,194 | 0.04 | 0.03 | 0.05 | 4.32E-08 | 3,087 | 1.05 | 0.95 | 1.15 | 3.99E-01 |
| Inflammation | Glycoprotein Acetyls | 22,194 | -0.03 | -0.04 | -0.02 | 3.56E-05 | 3,087 | 1.46 | 1.31 | 1.62 | 1.27E-11 |
| Lipoprotein subclasses | Concentration of Chylomicrons and Extremely Large VLDL Particles | 22,194 | -0.03 | -0.04 | -0.01 | 1.35E-04 | 3,087 | 1.53 | 1.38 | 1.71 | 1.13E-13 |
| Lipoprotein subclasses | Total Lipids in Chylomicrons and Extremely Large VLDL | 22,194 | -0.02 | -0.04 | -0.01 | 2.75E-04 | 3,087 | 1.51 | 1.36 | 1.68 | 4.01E-13 |
| Lipoprotein subclasses | Phospholipids in Chylomicrons and Extremely Large VLDL | 22,194 | -0.02 | -0.04 | -0.01 | 2.25E-04 | 3,087 | 1.53 | 1.37 | 1.71 | 1.61E-13 |
| Lipoprotein subclasses | Cholesterol in Chylomicrons and Extremely Large VLDL | 22,194 | -0.03 | -0.04 | -0.01 | 6.19E-05 | 3,087 | 1.51 | 1.35 | 1.68 | 4.54E-13 |
| Lipoprotein subclasses | Cholesteryl Esters in Chylomicrons and Extremely Large VLDL | 22,194 | -0.03 | -0.04 | -0.02 | 1.60E-05 | 3,087 | 1.49 | 1.34 | 1.65 | 1.37E-12 |
| Lipoprotein subclasses | Free Cholesterol in Chylomicrons and Extremely Large VLDL | 22,194 | -0.02 | -0.04 | -0.01 | 6.11E-04 | 3,087 | 1.51 | 1.36 | 1.68 | 3.84E-13 |
| Lipoprotein subclasses | Triglycerides in Chylomicrons and Extremely Large VLDL | 22,194 | -0.02 | -0.04 | -0.01 | 4.35E-04 | 3,087 | 1.51 | 1.35 | 1.68 | 6.21E-13 |
| Lipoprotein subclasses | Concentration of Very Large VLDL Particles | 22,194 | -0.03 | -0.04 | -0.02 | 1.05E-05 | 3,087 | 1.58 | 1.41 | 1.76 | 1.33E-14 |
| Lipoprotein subclasses | Total Lipids in Very Large VLDL | 22,194 | -0.03 | -0.04 | -0.02 | 1.42E-05 | 3,087 | 1.57 | 1.41 | 1.76 | 1.38E-14 |
| Lipoprotein subclasses | Phospholipids in Very Large VLDL | 22,194 | -0.03 | -0.04 | -0.01 | 9.93E-05 | 3,087 | 1.54 | 1.38 | 1.72 | 7.91E-14 |
| Lipoprotein subclasses | Cholesterol in Very Large VLDL | 22,194 | -0.02 | -0.03 | -0.01 | 1.22E-03 | 3,087 | 1.45 | 1.31 | 1.61 | 2.00E-11 |
| Lipoprotein subclasses | Cholesteryl Esters in Very Large VLDL | 22,194 | -0.02 | -0.03 | -0.01 | 2.60E-03 | 3,087 | 1.38 | 1.25 | 1.53 | 2.51E-09 |
| Lipoprotein subclasses | Free Cholesterol in Very Large VLDL | 22,194 | -0.02 | -0.04 | -0.01 | 8.79E-04 | 3,087 | 1.51 | 1.35 | 1.68 | 5.34E-13 |
| Lipoprotein subclasses | Triglycerides in Very Large VLDL | 22,194 | -0.03 | -0.04 | -0.02 | 1.28E-06 | 3,087 | 1.60 | 1.44 | 1.79 | 2.49E-15 |
| Lipoprotein subclasses | Concentration of Large VLDL Particles | 22,194 | -0.03 | -0.04 | -0.01 | 1.38E-04 | 3,087 | 1.52 | 1.36 | 1.69 | 3.64E-13 |
| Lipoprotein subclasses | Total Lipids in Large VLDL | 22,194 | -0.03 | -0.04 | -0.01 | 1.75E-04 | 3,087 | 1.51 | 1.35 | 1.68 | 6.17E-13 |
| Lipoprotein subclasses | Phospholipids in Large VLDL | 22,194 | -0.03 | -0.04 | -0.01 | 1.14E-04 | 3,087 | 1.52 | 1.37 | 1.70 | 3.07E-13 |
| Lipoprotein subclasses | Cholesterol in Large VLDL | 22,194 | -0.02 | -0.03 | -0.01 | 4.94E-03 | 3,087 | 1.42 | 1.28 | 1.58 | 2.13E-10 |
| Lipoprotein subclasses | Cholesteryl Esters in Large VLDL | 22,194 | -0.01 | -0.03 | 0.00 | 5.30E-02 | 3,087 | 1.33 | 1.20 | 1.47 | 1.50E-07 |
| Lipoprotein subclasses | Free Cholesterol in Large VLDL | 22,194 | -0.02 | -0.04 | -0.01 | 3.38E-04 | 3,087 | 1.51 | 1.35 | 1.68 | 6.17E-13 |
| Lipoprotein subclasses | Triglycerides in Large VLDL | 22,194 | -0.03 | -0.04 | -0.02 | 3.86E-05 | 3,087 | 1.53 | 1.37 | 1.70 | 2.66E-13 |
| Lipoprotein subclasses | Concentration of Medium VLDL Particles | 22,194 | -0.02 | -0.03 | 0.00 | 3.55E-02 | 3,087 | 1.25 | 1.13 | 1.38 | 2.55E-05 |
| Lipoprotein subclasses | Total Lipids in Medium VLDL | 22,194 | -0.02 | -0.03 | 0.00 | 1.27E-02 | 3,087 | 1.30 | 1.18 | 1.44 | 6.45E-07 |
| Lipoprotein subclasses | Phospholipids in Medium VLDL | 22,194 | -0.02 | -0.03 | 0.00 | 3.96E-02 | 3,087 | 1.23 | 1.11 | 1.35 | 9.60E-05 |
| Lipoprotein subclasses | Cholesterol in Medium VLDL | 22,194 | 0.00 | -0.02 | 0.01 | 7.07E-01 | 3,087 | 1.06 | 0.96 | 1.16 | 3.03E-01 |
| Lipoprotein subclasses | Cholesteryl Esters in Medium VLDL | 22,194 | 0.00 | -0.01 | 0.02 | 6.52E-01 | 3,087 | 0.97 | 0.88 | 1.07 | 6.46E-01 |
| Lipoprotein subclasses | Free Cholesterol in Medium VLDL | 22,194 | -0.01 | -0.02 | 0.00 | 1.50E-01 | 3,087 | 1.17 | 1.06 | 1.29 | 2.22E-03 |
| Lipoprotein subclasses | Triglycerides in Medium VLDL | 22,194 | -0.02 | -0.04 | -0.01 | 3.42E-04 | 3,087 | 1.45 | 1.31 | 1.61 | 2.21E-11 |
| Lipoprotein subclasses | Concentration of Small VLDL Particles | 22,194 | -0.01 | -0.03 | 0.00 | 4.15E-02 | 3,087 | 1.33 | 1.21 | 1.48 | 7.49E-08 |
| Lipoprotein subclasses | Total Lipids in Small VLDL | 22,194 | -0.02 | -0.03 | 0.00 | 2.88E-02 | 3,087 | 1.33 | 1.20 | 1.47 | 8.89E-08 |
| Lipoprotein subclasses | Phospholipids in Small VLDL | 22,194 | -0.01 | -0.03 | 0.00 | 8.41E-02 | 3,087 | 1.24 | 1.12 | 1.36 | 6.18E-05 |
| Lipoprotein subclasses | Cholesterol in Small VLDL | 22,194 | -0.01 | -0.03 | 0.00 | 8.95E-02 | 3,087 | 1.20 | 1.09 | 1.33 | 4.73E-04 |
| Lipoprotein subclasses | Cholesteryl Esters in Small VLDL | 22,194 | -0.01 | -0.03 | 0.00 | 7.01E-02 | 3,087 | 1.23 | 1.11 | 1.36 | 9.68E-05 |
| Lipoprotein subclasses | Free Cholesterol in Small VLDL | 22,194 | -0.01 | -0.02 | 0.00 | 1.44E-01 | 3,087 | 1.15 | 1.05 | 1.27 | 6.41E-03 |
| Lipoprotein subclasses | Triglycerides in Small VLDL | 22,194 | -0.02 | -0.03 | 0.00 | 1.50E-02 | 3,087 | 1.43 | 1.29 | 1.59 | 9.07E-11 |
| Lipoprotein subclasses | Concentration of Very Small VLDL Particles | 22,194 | -0.02 | -0.03 | 0.00 | 1.39E-02 | 3,087 | 1.17 | 1.06 | 1.29 | 2.58E-03 |
| Lipoprotein subclasses | Total Lipids in Very Small VLDL | 22,194 | -0.02 | -0.03 | 0.00 | 3.02E-02 | 3,087 | 1.17 | 1.06 | 1.29 | 3.22E-03 |
| Lipoprotein subclasses | Phospholipids in Very Small VLDL | 22,194 | -0.01 | -0.03 | 0.00 | 8.95E-02 | 3,087 | 1.19 | 1.08 | 1.31 | 1.08E-03 |
| Lipoprotein subclasses | Cholesterol in Very Small VLDL | 22,194 | -0.01 | -0.03 | 0.00 | 9.64E-02 | 3,087 | 1.05 | 0.95 | 1.16 | 4.21E-01 |
| Lipoprotein subclasses | Cholesteryl Esters in Very Small VLDL | 22,194 | -0.01 | -0.02 | 0.00 | 1.16E-01 | 3,087 | 1.01 | 0.91 | 1.12 | 8.83E-01 |
| Lipoprotein subclasses | Free Cholesterol in Very Small VLDL | 22,194 | -0.01 | -0.03 | 0.00 | 6.59E-02 | 3,087 | 1.14 | 1.03 | 1.25 | 1.68E-02 |
| Lipoprotein subclasses | Triglycerides in Very Small VLDL | 22,194 | -0.02 | -0.03 | -0.01 | 2.18E-03 | 3,087 | 1.39 | 1.26 | 1.55 | 6.92E-10 |
| Lipoprotein subclasses | Concentration of IDL Particles | 22,194 | -0.02 | -0.04 | -0.01 | 3.04E-03 | 3,087 | 1.06 | 0.96 | 1.17 | 2.63E-01 |
| Lipoprotein subclasses | Total Lipids in IDL | 22,194 | -0.02 | -0.04 | -0.01 | 1.09E-03 | 3,087 | 1.02 | 0.92 | 1.13 | 7.43E-01 |
| Lipoprotein subclasses | Phospholipids in IDL | 22,194 | -0.01 | -0.03 | 0.00 | 4.44E-02 | 3,087 | 1.01 | 0.91 | 1.11 | 9.17E-01 |
| Lipoprotein subclasses | Cholesterol in IDL | 22,194 | -0.02 | -0.04 | -0.01 | 1.09E-03 | 3,087 | 0.99 | 0.89 | 1.09 | 8.35E-01 |
| Lipoprotein subclasses | Cholesteryl Esters in IDL | 22,194 | -0.03 | -0.04 | -0.01 | 3.07E-04 | 3,087 | 0.99 | 0.89 | 1.09 | 8.35E-01 |
| Lipoprotein subclasses | Free Cholesterol in IDL | 22,194 | -0.02 | -0.03 | 0.00 | 2.77E-02 | 3,087 | 0.99 | 0.89 | 1.09 | 8.24E-01 |
| Lipoprotein subclasses | Triglycerides in IDL | 22,194 | -0.03 | -0.04 | -0.02 | 1.66E-05 | 3,087 | 1.37 | 1.24 | 1.51 | 3.60E-09 |
| Lipoprotein subclasses | Concentration of Large LDL Particles | 22,194 | 0.00 | -0.02 | 0.01 | 6.75E-01 | 3,087 | 1.05 | 0.96 | 1.16 | 3.18E-01 |
| Lipoprotein subclasses | Total Lipids in Large LDL | 22,194 | -0.02 | -0.04 | -0.01 | 9.94E-04 | 3,087 | 1.07 | 0.97 | 1.18 | 2.17E-01 |
| Lipoprotein subclasses | Phospholipids in Large LDL | 22,194 | -0.03 | -0.04 | -0.01 | 3.18E-04 | 3,087 | 1.07 | 0.97 | 1.18 | 1.92E-01 |
| Lipoprotein subclasses | Cholesterol in Large LDL | 22,194 | -0.02 | -0.03 | -0.01 | 3.94E-03 | 3,087 | 1.04 | 0.94 | 1.15 | 4.87E-01 |
| Lipoprotein subclasses | Cholesteryl Esters in Large LDL | 22,194 | -0.02 | -0.03 | -0.01 | 3.94E-03 | 3,087 | 1.06 | 0.96 | 1.17 | 2.78E-01 |
| Lipoprotein subclasses | Free Cholesterol in Large LDL | 22,194 | -0.02 | -0.03 | -0.01 | 4.76E-03 | 3,087 | 0.98 | 0.89 | 1.08 | 7.79E-01 |
| Lipoprotein subclasses | Triglycerides in Large LDL | 22,194 | -0.04 | -0.06 | -0.03 | 3.30E-09 | 3,087 | 1.41 | 1.27 | 1.56 | 1.09E-10 |
| Lipoprotein subclasses | Concentration of Medium LDL Particles | 22,194 | -0.03 | -0.04 | -0.01 | 4.13E-04 | 3,087 | 1.25 | 1.13 | 1.38 | 1.87E-05 |
| Lipoprotein subclasses | Total Lipids in Medium LDL | 22,194 | -0.03 | -0.04 | -0.02 | 4.30E-05 | 3,087 | 1.22 | 1.10 | 1.34 | 1.54E-04 |
| Lipoprotein subclasses | Phospholipids in Medium LDL | 22,194 | -0.03 | -0.04 | -0.01 | 1.18E-04 | 3,087 | 1.18 | 1.07 | 1.30 | 1.18E-03 |
| Lipoprotein subclasses | Cholesterol in Medium LDL | 22,194 | -0.03 | -0.04 | -0.02 | 8.46E-05 | 3,087 | 1.20 | 1.09 | 1.33 | 3.95E-04 |
| Lipoprotein subclasses | Cholesteryl Esters in Medium LDL | 22,194 | -0.03 | -0.05 | -0.02 | 1.72E-05 | 3,087 | 1.26 | 1.14 | 1.39 | 1.09E-05 |
| Lipoprotein subclasses | Free Cholesterol in Medium LDL | 22,194 | -0.02 | -0.03 | -0.01 | 5.52E-03 | 3,087 | 1.05 | 0.96 | 1.16 | 3.40E-01 |
| Lipoprotein subclasses | Triglycerides in Medium LDL | 22,194 | -0.04 | -0.05 | -0.03 | 4.07E-08 | 3,087 | 1.46 | 1.32 | 1.61 | 2.65E-12 |
| Lipoprotein subclasses | Concentration of Small LDL Particles | 22,194 | -0.01 | -0.03 | 0.00 | 4.81E-02 | 3,087 | 1.21 | 1.09 | 1.33 | 3.04E-04 |
| Lipoprotein subclasses | Total Lipids in Small LDL | 22,194 | -0.02 | -0.03 | -0.01 | 7.02E-03 | 3,087 | 1.19 | 1.08 | 1.31 | 9.17E-04 |
| Lipoprotein subclasses | Phospholipids in Small LDL | 22,194 | -0.01 | -0.02 | 0.00 | 1.28E-01 | 3,087 | 1.13 | 1.03 | 1.25 | 1.47E-02 |
| Lipoprotein subclasses | Cholesterol in Small LDL | 22,194 | -0.02 | -0.03 | -0.01 | 4.83E-03 | 3,087 | 1.16 | 1.06 | 1.28 | 3.32E-03 |
| Lipoprotein subclasses | Cholesteryl Esters in Small LDL | 22,194 | -0.02 | -0.04 | -0.01 | 9.94E-04 | 3,087 | 1.22 | 1.10 | 1.34 | 1.44E-04 |
| Lipoprotein subclasses | Free Cholesterol in Small LDL | 22,194 | -0.01 | -0.02 | 0.00 | 1.93E-01 | 3,087 | 1.01 | 0.92 | 1.12 | 8.03E-01 |
| Lipoprotein subclasses | Triglycerides in Small LDL | 22,194 | -0.03 | -0.04 | -0.02 | 2.73E-05 | 3,087 | 1.47 | 1.33 | 1.64 | 1.29E-12 |
| Lipoprotein subclasses | Concentration of Very Large HDL Particles | 22,194 | 0.02 | 0.01 | 0.03 | 1.86E-03 | 3,087 | 0.70 | 0.62 | 0.79 | 2.30E-08 |
| Lipoprotein subclasses | Total Lipids in Very Large HDL | 22,194 | 0.03 | 0.01 | 0.04 | 2.63E-05 | 3,087 | 0.66 | 0.59 | 0.75 | 1.78E-10 |
| Lipoprotein subclasses | Phospholipids in Very Large HDL | 22,194 | 0.03 | 0.02 | 0.04 | 1.36E-05 | 3,087 | 0.67 | 0.59 | 0.75 | 2.15E-10 |
| Lipoprotein subclasses | Cholesterol in Very Large HDL | 22,194 | 0.03 | 0.01 | 0.04 | 2.17E-05 | 3,087 | 0.64 | 0.57 | 0.73 | 8.44E-12 |
| Lipoprotein subclasses | Cholesteryl Esters in Very Large HDL | 22,194 | 0.03 | 0.01 | 0.04 | 1.72E-05 | 3,087 | 0.63 | 0.55 | 0.71 | 1.24E-12 |
| Lipoprotein subclasses | Free Cholesterol in Very Large HDL | 22,194 | 0.02 | 0.01 | 0.04 | 1.65E-04 | 3,087 | 0.73 | 0.65 | 0.82 | 1.81E-07 |
| Lipoprotein subclasses | Triglycerides in Very Large HDL | 22,194 | -0.02 | -0.04 | -0.01 | 3.07E-03 | 3,087 | 1.20 | 1.09 | 1.32 | 4.12E-04 |
| Lipoprotein subclasses | Concentration of Large HDL Particles | 22,194 | 0.01 | 0.00 | 0.02 | 6.25E-02 | 3,087 | 0.65 | 0.57 | 0.73 | 4.49E-11 |
| Lipoprotein subclasses | Total Lipids in Large HDL | 22,194 | 0.01 | 0.00 | 0.02 | 9.88E-02 | 3,087 | 0.66 | 0.59 | 0.75 | 1.78E-10 |
| Lipoprotein subclasses | Phospholipids in Large HDL | 22,194 | 0.01 | -0.01 | 0.02 | 3.25E-01 | 3,087 | 0.70 | 0.62 | 0.79 | 1.20E-08 |
| Lipoprotein subclasses | Cholesterol in Large HDL | 22,194 | 0.02 | 0.01 | 0.03 | 6.25E-03 | 3,087 | 0.62 | 0.55 | 0.70 | 5.34E-13 |
| Lipoprotein subclasses | Cholesteryl Esters in Large HDL | 22,194 | 0.02 | 0.01 | 0.03 | 3.37E-03 | 3,087 | 0.61 | 0.54 | 0.69 | 1.04E-13 |
| Lipoprotein subclasses | Free Cholesterol in Large HDL | 22,194 | 0.01 | 0.00 | 0.02 | 5.02E-02 | 3,087 | 0.67 | 0.59 | 0.76 | 5.10E-10 |
| Lipoprotein subclasses | Triglycerides in Large HDL | 22,194 | -0.03 | -0.05 | -0.02 | 3.67E-06 | 3,087 | 1.10 | 0.99 | 1.21 | 8.90E-02 |
| Lipoprotein subclasses | Concentration of Medium HDL Particles | 22,194 | -0.03 | -0.04 | -0.02 | 3.67E-06 | 3,087 | 0.91 | 0.82 | 1.02 | 1.18E-01 |
| Lipoprotein subclasses | Total Lipids in Medium HDL | 22,194 | -0.03 | -0.05 | -0.02 | 4.34E-07 | 3,087 | 0.95 | 0.85 | 1.06 | 3.94E-01 |
| Lipoprotein subclasses | Phospholipids in Medium HDL | 22,194 | -0.04 | -0.05 | -0.03 | 3.45E-08 | 3,087 | 1.00 | 0.90 | 1.11 | 9.74E-01 |
| Lipoprotein subclasses | Cholesterol in Medium HDL | 22,194 | -0.02 | -0.04 | -0.01 | 1.98E-04 | 3,087 | 0.85 | 0.76 | 0.95 | 6.99E-03 |
| Lipoprotein subclasses | Cholesteryl Esters in Medium HDL | 22,194 | -0.02 | -0.04 | -0.01 | 3.07E-04 | 3,087 | 0.85 | 0.76 | 0.94 | 3.93E-03 |
| Lipoprotein subclasses | Free Cholesterol in Medium HDL | 22,194 | -0.03 | -0.04 | -0.01 | 4.35E-05 | 3,087 | 0.89 | 0.80 | 1.00 | 6.36E-02 |
| Lipoprotein subclasses | Triglycerides in Medium HDL | 22,194 | -0.05 | -0.07 | -0.04 | 2.11E-13 | 3,087 | 1.37 | 1.24 | 1.52 | 4.31E-09 |
| Lipoprotein subclasses | Concentration of Small HDL Particles | 22,194 | -0.06 | -0.07 | -0.04 | 2.47E-15 | 3,087 | 1.19 | 1.08 | 1.31 | 9.17E-04 |
| Lipoprotein subclasses | Total Lipids in Small HDL | 22,194 | -0.06 | -0.08 | -0.05 | 1.91E-19 | 3,087 | 1.26 | 1.14 | 1.39 | 1.49E-05 |
| Lipoprotein subclasses | Phospholipids in Small HDL | 22,194 | -0.07 | -0.08 | -0.05 | 2.49E-20 | 3,087 | 1.25 | 1.13 | 1.38 | 2.11E-05 |
| Lipoprotein subclasses | Cholesterol in Small HDL | 22,194 | -0.05 | -0.07 | -0.04 | 5.21E-14 | 3,087 | 1.14 | 1.03 | 1.26 | 1.26E-02 |
| Lipoprotein subclasses | Cholesteryl Esters in Small HDL | 22,194 | -0.05 | -0.07 | -0.04 | 1.46E-12 | 3,087 | 1.12 | 1.02 | 1.24 | 3.32E-02 |
| Lipoprotein subclasses | Free Cholesterol in Small HDL | 22,194 | -0.06 | -0.07 | -0.04 | 2.72E-15 | 3,087 | 1.18 | 1.07 | 1.31 | 1.32E-03 |
| Lipoprotein subclasses | Triglycerides in Small HDL | 22,194 | -0.04 | -0.06 | -0.03 | 8.54E-11 | 3,087 | 1.57 | 1.41 | 1.75 | 1.37E-14 |
| Relative lipoprotein lipid concentrations | Phospholipids to Total Lipids in Chylomicrons and Extremely Large VLDL percentage | 22,194 | 0.01 | -0.04 | 0.07 | 4.07E-03 | 3,087 | 1.01 | 0.99 | 1.04 | 4.13E-01 |
| Relative lipoprotein lipid concentrations | Cholesterol to Total Lipids in Chylomicrons and Extremely Large VLDL percentage | 22,194 | -0.07 | -0.22 | 0.09 | 5.13E-01 | 3,087 | 0.98 | 0.97 | 0.99 | 8.59E-06 |
| Relative lipoprotein lipid concentrations | Cholesteryl Esters to Total Lipids in Chylomicrons and Extremely Large VLDL percentage | 22,194 | -0.12 | -0.23 | -0.01 | 2.70E-01 | 3,087 | 0.98 | 0.97 | 1.00 | 9.82E-04 |
| Relative lipoprotein lipid concentrations | Free Cholesterol to Total Lipids in Chylomicrons and Extremely Large VLDL percentage | 22,194 | 0.06 | 0.00 | 0.11 | 2.38E-04 | 3,087 | 0.94 | 0.92 | 0.97 | 4.60E-09 |
| Relative lipoprotein lipid concentrations | Triglycerides to Total Lipids in Chylomicrons and Extremely Large VLDL percentage | 22,194 | 0.05 | -0.12 | 0.23 | 4.80E-01 | 3,087 | 1.01 | 1.00 | 1.02 | 5.10E-04 |
| Relative lipoprotein lipid concentrations | Phospholipids to Total Lipids in Very Large VLDL percentage | 22,194 | 0.01 | -0.02 | 0.05 | 2.93E-03 | 3,087 | 1.03 | 0.99 | 1.08 | 9.74E-01 |
| Relative lipoprotein lipid concentrations | Cholesterol to Total Lipids in Very Large VLDL percentage | 22,194 | 0.23 | 0.13 | 0.33 | 6.57E-07 | 3,087 | 0.95 | 0.93 | 0.96 | 2.74E-12 |
| Relative lipoprotein lipid concentrations | Cholesteryl Esters to Total Lipids in Very Large VLDL percentage | 22,194 | 0.16 | 0.07 | 0.24 | 3.11E-05 | 3,087 | 0.93 | 0.92 | 0.95 | 1.04E-11 |
| Relative lipoprotein lipid concentrations | Free Cholesterol to Total Lipids in Very Large VLDL percentage | 22,194 | 0.07 | 0.05 | 0.10 | 1.92E-13 | 3,087 | 0.81 | 0.75 | 0.87 | 7.03E-11 |
| Relative lipoprotein lipid concentrations | Triglycerides to Total Lipids in Very Large VLDL percentage | 22,194 | -0.24 | -0.35 | -0.14 | 5.32E-07 | 3,087 | 1.04 | 1.03 | 1.06 | 6.92E-10 |
| Relative lipoprotein lipid concentrations | Phospholipids to Total Lipids in Large VLDL percentage | 22,194 | -0.08 | -0.12 | -0.03 | 1.41E-03 | 3,087 | 1.11 | 1.07 | 1.17 | 3.09E-07 |
| Relative lipoprotein lipid concentrations | Cholesterol to Total Lipids in Large VLDL percentage | 22,194 | 0.11 | 0.05 | 0.17 | 2.25E-04 | 3,087 | 0.94 | 0.92 | 0.97 | 3.26E-06 |
| Relative lipoprotein lipid concentrations | Cholesteryl Esters to Total Lipids in Large VLDL percentage | 22,194 | 0.11 | 0.07 | 0.16 | 7.99E-07 | 3,087 | 0.90 | 0.87 | 0.93 | 2.43E-10 |
| Relative lipoprotein lipid concentrations | Free Cholesterol to Total Lipids in Large VLDL percentage | 22,194 | 0.00 | -0.02 | 0.01 | 5.08E-01 | 3,087 | 1.07 | 0.99 | 1.16 | 4.17E-01 |
| Relative lipoprotein lipid concentrations | Triglycerides to Total Lipids in Large VLDL percentage | 22,194 | -0.03 | -0.12 | 0.05 | 7.01E-02 | 3,087 | 1.01 | 0.99 | 1.03 | 1.32E-01 |
| Relative lipoprotein lipid concentrations | Phospholipids to Total Lipids in Medium VLDL percentage | 22,194 | 0.01 | -0.01 | 0.04 | 2.91E-01 | 3,087 | 0.89 | 0.84 | 0.95 | 3.08E-04 |
| Relative lipoprotein lipid concentrations | Cholesterol to Total Lipids in Medium VLDL percentage | 22,194 | 0.16 | 0.08 | 0.24 | 1.38E-04 | 3,087 | 0.95 | 0.94 | 0.97 | 3.03E-09 |
| Relative lipoprotein lipid concentrations | Cholesteryl Esters to Total Lipids in Medium VLDL percentage | 22,194 | 0.13 | 0.07 | 0.19 | 3.92E-05 | 3,087 | 0.93 | 0.91 | 0.95 | 2.70E-10 |
| Relative lipoprotein lipid concentrations | Free Cholesterol to Total Lipids in Medium VLDL percentage | 22,194 | 0.03 | 0.01 | 0.05 | 4.37E-03 | 3,087 | 0.86 | 0.81 | 0.91 | 3.15E-06 |
| Relative lipoprotein lipid concentrations | Triglycerides to Total Lipids in Medium VLDL percentage | 22,194 | -0.17 | -0.28 | -0.07 | 1.09E-03 | 3,087 | 1.04 | 1.03 | 1.05 | 3.58E-08 |
| Relative lipoprotein lipid concentrations | Phospholipids to Total Lipids in Small VLDL percentage | 22,194 | 0.02 | 0.00 | 0.05 | 9.68E-02 | 3,087 | 0.88 | 0.84 | 0.93 | 2.87E-06 |
| Relative lipoprotein lipid concentrations | Cholesterol to Total Lipids in Small VLDL percentage | 22,194 | 0.02 | -0.04 | 0.08 | 5.08E-01 | 3,087 | 0.96 | 0.94 | 0.98 | 2.13E-04 |
| Relative lipoprotein lipid concentrations | Cholesteryl Esters to Total Lipids in Small VLDL percentage | 22,194 | 0.00 | -0.03 | 0.04 | 8.14E-01 | 3,087 | 0.95 | 0.92 | 0.99 | 1.15E-02 |
| Relative lipoprotein lipid concentrations | Free Cholesterol to Total Lipids in Small VLDL percentage | 22,194 | 0.02 | -0.01 | 0.05 | 1.84E-01 | 3,087 | 0.88 | 0.84 | 0.93 | 1.35E-06 |
| Relative lipoprotein lipid concentrations | Triglycerides to Total Lipids in Small VLDL percentage | 22,194 | -0.05 | -0.13 | 0.04 | 3.42E-01 | 3,087 | 1.03 | 1.02 | 1.05 | 5.16E-05 |
| Relative lipoprotein lipid concentrations | Phospholipids to Total Lipids in Very Small VLDL percentage | 22,194 | 0.02 | 0.01 | 0.03 | 1.31E-04 | 3,087 | 1.21 | 1.07 | 1.37 | 3.69E-03 |
| Relative lipoprotein lipid concentrations | Cholesterol to Total Lipids in Very Small VLDL percentage | 22,194 | 0.03 | -0.03 | 0.08 | 5.02E-01 | 3,087 | 0.93 | 0.91 | 0.95 | 3.54E-08 |
| Relative lipoprotein lipid concentrations | Cholesteryl Esters to Total Lipids in Very Small VLDL percentage | 22,194 | 0.02 | -0.03 | 0.07 | 5.33E-01 | 3,087 | 0.92 | 0.90 | 0.95 | 5.58E-09 |
| Relative lipoprotein lipid concentrations | Free Cholesterol to Total Lipids in Very Small VLDL percentage | 22,194 | 0.01 | 0.00 | 0.01 | 2.93E-01 | 3,087 | 0.81 | 0.69 | 0.94 | 6.67E-02 |
| Relative lipoprotein lipid concentrations | Triglycerides to Total Lipids in Very Small VLDL percentage | 22,194 | -0.05 | -0.10 | 0.00 | 9.57E-02 | 3,087 | 1.08 | 1.05 | 1.11 | 5.33E-08 |
| Relative lipoprotein lipid concentrations | Phospholipids to Total Lipids in IDL percentage | 22,194 | 0.06 | 0.04 | 0.07 | 1.08E-17 | 3,087 | 0.92 | 0.82 | 1.02 | 1.50E-01 |
| Relative lipoprotein lipid concentrations | Cholesterol to Total Lipids in IDL percentage | 22,194 | -0.04 | -0.08 | 0.00 | 6.81E-03 | 3,087 | 0.93 | 0.90 | 0.96 | 2.91E-04 |
| Relative lipoprotein lipid concentrations | Cholesteryl Esters to Total Lipids in IDL percentage | 22,194 | -0.07 | -0.10 | -0.03 | 7.44E-06 | 3,087 | 0.93 | 0.89 | 0.96 | 9.50E-04 |
| Relative lipoprotein lipid concentrations | Free Cholesterol to Total Lipids in IDL percentage | 22,194 | 0.02 | 0.01 | 0.04 | 1.71E-03 | 3,087 | 0.87 | 0.79 | 0.95 | 8.73E-03 |
| Relative lipoprotein lipid concentrations | Triglycerides to Total Lipids in IDL percentage | 22,194 | -0.01 | -0.04 | 0.02 | 3.91E-01 | 3,087 | 1.13 | 1.08 | 1.18 | 9.07E-08 |
| Relative lipoprotein lipid concentrations | Phospholipids to Total Lipids in Large LDL percentage | 22,194 | -0.01 | -0.02 | 0.00 | 1.39E-01 | 3,087 | 1.02 | 0.89 | 1.17 | 8.39E-01 |
| Relative lipoprotein lipid concentrations | Cholesterol to Total Lipids in Large LDL percentage | 22,194 | 0.04 | 0.01 | 0.06 | 1.27E-03 | 3,087 | 0.82 | 0.76 | 0.87 | 2.34E-09 |
| Relative lipoprotein lipid concentrations | Cholesteryl Esters to Total Lipids in Large LDL percentage | 22,194 | 0.03 | 0.01 | 0.04 | 2.68E-03 | 3,087 | 0.94 | 0.86 | 1.03 | 2.64E-01 |
| Relative lipoprotein lipid concentrations | Free Cholesterol to Total Lipids in Large LDL percentage | 22,194 | 0.01 | -0.01 | 0.02 | 3.81E-01 | 3,087 | 0.76 | 0.70 | 0.82 | 2.43E-10 |
| Relative lipoprotein lipid concentrations | Triglycerides to Total Lipids in Large LDL percentage | 22,194 | -0.03 | -0.05 | -0.01 | 1.46E-02 | 3,087 | 1.19 | 1.12 | 1.27 | 3.34E-08 |
| Relative lipoprotein lipid concentrations | Triglyceride | 22,194 | 0.03 | 0.01 | 0.04 | 5.86E-04 | 3,087 | 0.76 | 0.69 | 0.84 | 1.89E-07 |
| Relative lipoprotein lipid concentrations | Cholesterol to Total Lipids in Medium LDL percentage | 22,194 | -0.01 | -0.03 | 0.02 | 3.60E-01 | 3,087 | 0.97 | 0.90 | 1.03 | 4.21E-01 |
| Relative lipoprotein lipid concentrations | Cholesteryl Esters to Total Lipids in Medium LDL percentage | 22,194 | -0.06 | -0.08 | -0.03 | 5.65E-05 | 3,087 | 1.22 | 1.15 | 1.29 | 2.22E-11 |
| Relative lipoprotein lipid concentrations | Free Cholesterol to Total Lipids in Medium LDL percentage | 22,194 | 0.05 | 0.03 | 0.08 | 5.52E-06 | 3,087 | 0.79 | 0.75 | 0.84 | 2.05E-14 |
| Relative lipoprotein lipid concentrations | Triglycerides to Total Lipids in Medium LDL percentage | 22,194 | -0.01 | -0.03 | 0.01 | 2.17E-01 | 3,087 | 1.16 | 1.08 | 1.24 | 3.22E-05 |
| Relative lipoprotein lipid concentrations | Phospholipids to Total Lipids in Small LDL percentage | 22,194 | 0.07 | 0.05 | 0.10 | 2.26E-08 | 3,087 | 0.87 | 0.82 | 0.92 | 5.16E-06 |
| Relative lipoprotein lipid concentrations | Cholesterol to Total Lipids in Small LDL percentage | 22,194 | -0.04 | -0.07 | -0.01 | 2.10E-03 | 3,087 | 0.97 | 0.92 | 1.02 | 2.51E-01 |
| Relative lipoprotein lipid concentrations | Cholesteryl Esters to Total Lipids in Small LDL percentage | 22,194 | -0.08 | -0.10 | -0.05 | 4.48E-07 | 3,087 | 1.12 | 1.07 | 1.18 | 3.65E-06 |
| Relative lipoprotein lipid concentrations | Free Cholesterol to Total Lipids in Small LDL percentage | 22,194 | 0.04 | 0.01 | 0.06 | 2.03E-03 | 3,087 | 0.85 | 0.81 | 0.89 | 2.02E-09 |
| Relative lipoprotein lipid concentrations | Triglycerides to Total Lipids in Small LDL percentage | 22,194 | -0.03 | -0.05 | -0.01 | 1.18E-02 | 3,087 | 1.19 | 1.12 | 1.26 | 8.36E-09 |
| Relative lipoprotein lipid concentrations | Phospholipids to Total Lipids in Very Large HDL percentage | 22,194 | 0.17 | 0.10 | 0.25 | 1.15E-05 | 3,087 | 0.96 | 0.95 | 0.98 | 3.36E-09 |
| Relative lipoprotein lipid concentrations | Cholesterol to Total Lipids in Very Large HDL percentage | 22,194 | -0.06 | -0.12 | 0.00 | 1.08E-01 | 3,087 | 1.02 | 1.00 | 1.04 | 1.04E-01 |
| Relative lipoprotein lipid concentrations | Cholesteryl Esters to Total Lipids in Very Large HDL percentage | 22,194 | 0.01 | -0.03 | 0.06 | 6.12E-01 | 3,087 | 0.96 | 0.93 | 0.98 | 7.51E-04 |
| Relative lipoprotein lipid concentrations | Free Cholesterol to Total Lipids in Very Large HDL percentage | 22,194 | -0.07 | -0.12 | -0.03 | 3.78E-04 | 3,087 | 1.10 | 1.06 | 1.13 | 5.36E-11 |
| Relative lipoprotein lipid concentrations | Triglycerides to Total Lipids in Very Large HDL percentage | 22,194 | -0.11 | -0.16 | -0.07 | 1.70E-09 | 3,087 | 1.07 | 1.04 | 1.11 | 5.87E-14 |
| Relative lipoprotein lipid concentrations | Phospholipids to Total Lipids in Large HDL percentage | 22,194 | -0.10 | -0.14 | -0.07 | 5.19E-10 | 3,087 | 1.16 | 1.12 | 1.21 | 1.13E-13 |
| Relative lipoprotein lipid concentrations | Cholesterol to Total Lipids in Large HDL percentage | 22,194 | 0.19 | 0.13 | 0.26 | 2.51E-13 | 3,087 | 0.92 | 0.90 | 0.94 | 1.52E-17 |
| Relative lipoprotein lipid concentrations | Cholesteryl Esters to Total Lipids in Large HDL percentage | 22,194 | 0.18 | 0.12 | 0.23 | 7.01E-14 | 3,087 | 0.91 | 0.89 | 0.93 | 1.40E-17 |
| Relative lipoprotein lipid concentrations | Free Cholesterol to Total Lipids in Large HDL percentage | 22,194 | 0.02 | 0.00 | 0.03 | 2.19E-03 | 3,087 | 0.86 | 0.79 | 0.93 | 8.55E-04 |
| Relative lipoprotein lipid concentrations | Triglycerides to Total Lipids in Large HDL percentage | 22,194 | -0.09 | -0.13 | -0.05 | 1.87E-08 | 3,087 | 1.11 | 1.07 | 1.14 | 2.61E-12 |
| Relative lipoprotein lipid concentrations | Phospholipids to Total Lipids in Medium HDL percentage | 22,194 | -0.01 | -0.03 | 0.00 | 6.38E-02 | 3,087 | 1.37 | 1.26 | 1.49 | 1.33E-12 |
| Relative lipoprotein lipid concentrations | Cholesterol to Total Lipids in Medium HDL percentage | 22,194 | 0.06 | 0.03 | 0.10 | 4.90E-04 | 3,087 | 0.87 | 0.84 | 0.90 | 7.61E-13 |
| Relative lipoprotein lipid concentrations | Cholesteryl Esters to Total Lipids in Medium HDL percentage | 22,194 | 0.06 | 0.03 | 0.09 | 3.36E-05 | 3,087 | 0.86 | 0.83 | 0.90 | 3.60E-12 |
| Relative lipoprotein lipid concentrations | Free Cholesterol to Total Lipids in Medium HDL percentage | 22,194 | 0.00 | -0.01 | 0.01 | 6.54E-01 | 3,087 | 0.68 | 0.58 | 0.80 | 1.21E-05 |
| Relative lipoprotein lipid concentrations | Triglycerides to Total Lipids in Medium HDL percentage | 22,194 | -0.05 | -0.07 | -0.03 | 1.36E-05 | 3,087 | 1.23 | 1.16 | 1.30 | 1.32E-11 |
| Relative lipoprotein lipid concentrations | Phospholipids to Total Lipids in Small HDL percentage | 22,194 | -0.02 | -0.04 | 0.00 | 2.34E-02 | 3,087 | 1.01 | 0.93 | 1.10 | 8.24E-01 |
| Relative lipoprotein lipid concentrations | Cholesterol to Total Lipids in Small HDL percentage | 22,194 | 0.05 | 0.02 | 0.07 | 1.64E-04 | 3,087 | 0.87 | 0.82 | 0.92 | 1.48E-06 |
| Relative lipoprotein lipid concentrations | Cholesteryl Esters to Total Lipids in Small HDL percentage | 22,194 | 0.04 | 0.01 | 0.06 | 2.40E-03 | 3,087 | 0.89 | 0.84 | 0.94 | 4.09E-05 |
| Relative lipoprotein lipid concentrations | Free Cholesterol to Total Lipids in Small HDL percentage | 22,194 | 0.01 | 0.00 | 0.02 | 3.80E-02 | 3,087 | 0.73 | 0.59 | 0.91 | 5.66E-03 |
| Relative lipoprotein lipid concentrations | Triglycerides to Total Lipids in Small HDL percentage | 22,194 | -0.03 | -0.04 | -0.01 | 1.42E-03 | 3,087 | 1.36 | 1.25 | 1.47 | 2.68E-12 |

*Definition of abbreviations:* n, the number of participants used in observational test; beta, effect size; OR, odds ratio; CI Lower 95%, lower bound of the 95% confidence interval; CI Upper 95%, upper limit of the 95% confidence interval;FDR p, p value after FDR corrected.

**Supplementary Table S9: Fatty acid markers of DFP1 and NAFLD were jointly significantly associated**

| **Serum metabolic biomarkers** | | **DFP1** | | | | | **NAFLD** | | | | |
| --- | --- | --- | --- | --- | --- | --- | --- | --- | --- | --- | --- |
| **Group** | **Description** | **n** | **beta** | **CI Lower 95%** | **CI Upper 95%** | **FDR p** | **n** | **OR** | **CI Lower 95%** | **CI Upper 95%** | **FDR p** |
| Fatty acids | Total Fatty Acids | 22,194 | -0.03 | -0.05 | -0.02 | 4.95E-06 | 3,087 | 1.33 | 1.21 | 1.47 | 3.22E-08 |
| Fatty acids | Degree of Unsaturation | 22,194 | 0.01 | 0.01 | 0.01 | 1.07E-69 | 3,087 | 0.01 | 0.00 | 0.02 | 1.88E-12 |
| Fatty acids | Monounsaturated Fatty Acids | 22,194 | -0.05 | -0.06 | -0.03 | 3.03E-11 | 3,087 | 1.45 | 1.31 | 1.60 | 6.70E-12 |
| Fatty acids | Saturated Fatty Acids | 22,194 | -0.06 | -0.07 | -0.05 | 4.12E-18 | 3,087 | 1.43 | 1.30 | 1.59 | 8.44E-12 |
| Fatty acids | Omega-6 Fatty Acids to Total Fatty Acids percentage | 22,194 | 0.22 | 0.18 | 0.27 | 5.80E-25 | 3,087 | 0.86 | 0.84 | 0.89 | 2.65E-20 |
| Fatty acids | Polyunsaturated Fatty Acids to Total Fatty Acids percentage | 22,194 | 0.37 | 0.33 | 0.42 | 1.40E-65 | 3,087 | 0.86 | 0.84 | 0.89 | 2.14E-21 |
| Fatty acids | Monounsaturated Fatty Acids to Total Fatty Acids percentage | 22,194 | -0.14 | -0.17 | -0.11 | 3.52E-17 | 3,087 | 1.18 | 1.13 | 1.23 | 3.17E-14 |
| Fatty acids | Saturated Fatty Acids to Total Fatty Acids percentage | 22,194 | -0.23 | -0.25 | -0.20 | 1.74E-71 | 3,087 | 1.24 | 1.18 | 1.30 | 1.19E-14 |
| Fatty acids | Linoleic Acid to Total Fatty Acids percentage | 22,194 | 0.19 | 0.15 | 0.23 | 1.56E-18 | 3,087 | 0.86 | 0.83 | 0.89 | 2.54E-18 |
| Fatty acids | Docosahexaenoic Acid to Total Fatty Acids percentage | 22,194 | 0.06 | 0.06 | 0.07 | 2.89E-43 | 3,087 | 0.70 | 0.59 | 0.82 | 4.04E-06 |
| Fatty acids | Polyunsaturated Fatty Acids to Monounsaturated Fatty Acids ratio | 22,194 | 0.03 | 0.02 | 0.03 | 5.30E-35 | 3,087 | 0.21 | 0.15 | 0.29 | 1.63E-18 |

*Definition of abbreviations:* n, the number of participants used in observational test; beta, effect size; OR, odds ratio; CI Lower 95%, lower bound of the 95% confidence interval; CI Upper 95%, upper limit of the 95% confidence interval;FDR p, p value after FDR corrected.

**Supplementary Table S10: Indirect effect mediated by serum fatty acids in the effect of DFPs on NAFLD**

|  |  | **NDE** | | | | | **NIE** | | | |  |
| --- | --- | --- | --- | --- | --- | --- | --- | --- | --- | --- | --- |
| **Group** | **Description** | **n** | **OR** | **CI Lower 95%** | **CI Upper 95%** | **p** | **OR** | **CI Lower 95%** | **CI Upper 95%** | **p** | **PM** |
| DFP1 | Total Fatty Acids | 3,087 | 0.82 | 0.74 | 0.92 | 6.16E-04 | 1.00 | 0.99 | 1.01 | 0.71 |  |
|  | Degree of Unsaturation | 3,087 | 0.86 | 0.77 | 0.96 | 6.82E-03 | 0.96 | 0.94 | 0.98 | 4.53E-05 | 21.9% |
|  | Monounsaturated Fatty Acids | 3,087 | 0.83 | 0.74 | 0.93 | 7.79E-04 | 0.99 | 0.98 | 1.01 | 0.40 |  |
|  | Saturated Fatty Acids | 3,087 | 0.83 | 0.75 | 0.93 | 1.42E-03 | 0.98 | 0.97 | 1.00 | 0.04 | 7.9% |
|  | Omega-6 Fatty Acids to Total Fatty Acids percentage | 3,087 | 0.85 | 0.76 | 0.94 | 2.46E-03 | 0.97 | 0.95 | 0.99 | 1.11E-02 | 14.3% |
|  | Polyunsaturated Fatty Acids to Total Fatty Acids percentage | 3,087 | 0.87 | 0.78 | 0.96 | 0.01 | 0.95 | 0.93 | 0.98 | 9.02E-05 | 25.1% |
|  | Monounsaturated Fatty Acids to Total Fatty Acids percentage | 3,087 | 0.83 | 0.75 | 0.93 | 1.03E-03 | 0.99 | 0.97 | 1.01 | 0.21 |  |
|  | Saturated Fatty Acids to Total Fatty Acids percentage | 3,087 | 0.87 | 0.78 | 0.97 | 0.01 | 0.95 | 0.93 | 0.97 | 5.49E-07 | 27.8% |
|  | Linoleic Acid to Total Fatty Acids percentage | 3,087 | 0.85 | 0.76 | 0.94 | 2.73E-03 | 0.97 | 0.95 | 0.99 | 3.79E-03 | 15.6% |
|  | Docosahexaenoic Acid to Total Fatty Acids percentage | 3,087 | 0.84 | 0.75 | 0.94 | 1.94E-03 | 0.98 | 0.97 | 0.99 | 3.51E-03 | 10.0% |
|  | Polyunsaturated Fatty Acids to Monounsaturated Fatty Acids ratio | 3,087 | 0.85 | 0.76 | 0.95 | 3.19E-03 | 0.97 | 0.95 | 0.99 | 5.67E-03 | 15.9% |
| DFP2 | Omega-3 Fatty Acids to Total Fatty Acids percentage | 3,087 | 1.14 | 1.03 | 1.26 | 8.07E-03 | 0.98 | 0.96 | 1.00 | 0.05 | -18.9% |
|  | Omega-6 Fatty Acids to Total Fatty Acids percentage | 3,087 | 1.11 | 1.01 | 1.22 | 0.03 | 1.01 | 0.99 | 1.03 | 0.43 |  |
|  | Polyunsaturated Fatty Acids to Total Fatty Acids percentage | 3,087 | 1.16 | 1.05 | 1.27 | 2.14E-03 | 0.97 | 0.94 | 0.99 | 2.38E-03 | -31.0% |
|  | Monounsaturated Fatty Acids to Total Fatty Acids percentage | 3,087 | 1.16 | 1.05 | 1.27 | 1.99E-03 | 0.96 | 0.94 | 0.98 | 3.27E-04 | -33.8% |
|  | Saturated Fatty Acids to Total Fatty Acids percentage | 3,087 | 1.12 | 1.02 | 1.24 | 0.02 | 1.00 | 0.98 | 1.01 | 0.70 |  |
|  | Linoleic Acid to Total Fatty Acids percentage | 3,087 | 1.07 | 0.97 | 1.18 | 0.14 | 1.04 | 1.02 | 1.06 | 1.59E-04 |  |
|  | Docosahexaenoic Acid to Total Fatty Acids percentage | 3,087 | 1.18 | 1.06 | 1.29 | 1.17E-03 | 0.95 | 0.93 | 0.97 | 2.54E-05 | -45.4% |
|  | Polyunsaturated Fatty Acids to Monounsaturated Fatty Acids ratio | 3,087 | 1.17 | 1.06 | 1.28 | 1.27E-03 | 0.96 | 0.94 | 0.98 | 2.13E-04 | -37.9% |
|  | Omega-6 Fatty Acids to Omega-3 Fatty Acids ratio | 3,087 | 1.12 | 1.01 | 1.23 | 0.03 | 1.00 | 0.98 | 1.02 | 0.90 |  |
| DFP3 | Omega-3 Fatty Acids to Total Fatty Acids percentage | 3,087 | 1.01 | 0.92 | 1.11 | 0.80 | 1.01 | 1.00 | 1.02 | 0.19 |  |
|  | Omega-6 Fatty Acids to Total Fatty Acids percentage | 3,087 | 1.02 | 0.93 | 1.12 | 0.72 | 1.00 | 0.98 | 1.02 | 0.90 |  |
|  | Polyunsaturated Fatty Acids to Total Fatty Acids percentage | 3,087 | 1.00 | 0.91 | 1.10 | 1.00 | 1.02 | 1.00 | 1.04 | 0.08 |  |
|  | Monounsaturated Fatty Acids to Total Fatty Acids percentage | 3,087 | 1.01 | 0.93 | 1.11 | 0.78 | 1.01 | 0.99 | 1.02 | 0.51 |  |
|  | Saturated Fatty Acids to Total Fatty Acids percentage | 3,087 | 1.00 | 0.91 | 1.10 | 1.00 | 1.02 | 1.00 | 1.03 | 0.03 |  |
|  | Linoleic Acid to Total Fatty Acids percentage | 3,087 | 1.03 | 0.94 | 1.13 | 0.51 | 0.99 | 0.97 | 1.00 | 0.15 |  |
|  | Docosahexaenoic Acid to Total Fatty Acids percentage | 3,087 | 1.00 | 0.92 | 1.10 | 0.95 | 1.02 | 1.00 | 1.03 | 0.01 |  |
|  | Polyunsaturated Fatty Acids to Monounsaturated Fatty Acids ratio | 3,087 | 1.01 | 0.92 | 1.10 | 0.91 | 1.01 | 0.99 | 1.03 | 0.17 |  |
|  | Omega-6 Fatty Acids to Omega-3 Fatty Acids ratio | 3,087 | 1.02 | 0.93 | 1.12 | 0.68 | 1.00 | 0.99 | 1.00 | 0.65 |  |
| DFP4 | Omega-3 Fatty Acids to Total Fatty Acids percentage | 3,087 | 0.97 | 0.88 | 1.07 | 0.48 | 1.00 | 0.99 | 1.00 | 0.50 |  |
|  | Omega-6 Fatty Acids to Total Fatty Acids percentage | 3,087 | 0.99 | 0.90 | 1.10 | 0.86 | 0.97 | 0.95 | 0.99 | 0.01 |  |
|  | Polyunsaturated Fatty Acids to Total Fatty Acids percentage | 3,087 | 1.00 | 0.90 | 1.10 | 0.94 | 0.97 | 0.95 | 0.99 | 3.88E-03 |  |
|  | Monounsaturated Fatty Acids to Total Fatty Acids percentage | 3,087 | 0.96 | 0.88 | 1.06 | 0.45 | 1.00 | 0.98 | 1.02 | 0.96 |  |
|  | Saturated Fatty Acids to Total Fatty Acids percentage | 3,087 | 1.01 | 0.92 | 1.12 | 0.78 | 0.95 | 0.93 | 0.97 | 1.45E-06 |  |
|  | Linoleic Acid to Total Fatty Acids percentage | 3,087 | 1.01 | 0.91 | 1.11 | 0.89 | 0.96 | 0.94 | 0.98 | 5.78E-05 |  |
|  | Docosahexaenoic Acid to Total Fatty Acids percentage | 3,087 | 0.97 | 0.88 | 1.07 | 0.53 | 0.99 | 0.98 | 1.00 | 0.23 |  |
|  | Polyunsaturated Fatty Acids to Monounsaturated Fatty Acids ratio | 3,087 | 0.98 | 0.89 | 1.08 | 0.65 | 0.99 | 0.97 | 1.01 | 0.16 |  |
|  | Omega-6 Fatty Acids to Omega-3 Fatty Acids ratio | 3,087 | 0.96 | 0.88 | 1.06 | 0.46 | 1.00 | 1.00 | 1.00 | 0.94 |  |

*Definition of abbreviations:* n, the number of participants used in observational test; OR, odds ratio; CI Lower 95%, lower bound of the 95% confidence interval; CI Upper 95%, upper limit of the 95% confidence interval; p, p value; NDE, natural direct effect; NIE, natural indirect effect; PM, proportion of the effect of DFP on NAFLD that goes through fatty acids.
